# A Novel Isotope-labeled Small Molecule Probe CC12 for Anti-glioma via Suppressing LYN-mediated Progression and Activating Apoptosis Pathways

**DOI:** 10.7150/ijbs.82266

**Published:** 2023-06-19

**Authors:** Hsu-Shan Huang, I-Tsang Chiang, Bashir Lawal, Yueh-Shan Weng, Long-Bin Jeng, Yu-Cheng Kuo, Yu-Chang Liu, Fei-Ting Hsu

**Affiliations:** 1 PhD Program for Cancer Molecular Biology and Drug Discovery, College of Medical Science and Technology, Taipei Medical University, Taipei 110, Taiwan; and Academia Sinica, Taipei 115, Taiwan, R.O.C.; 2 Graduate Institute for Cancer Biology & Drug Discovery, College of Medical Science and Technology, Taipei Medical University, Taipei 110, Taiwan, R.O.C.; 3 Department of Radiation Oncology, Show Chwan Memorial Hospital, Changhua 500, Taiwan, R.O.C.; 4 Department of Radiation Oncology, Chang Bing Show Chwan Memorial Hospital, Lukang, Changhua 505, Taiwan, R.O.C.; 5 Department of Medical Imaging and Radiological Sciences, Central Taiwan University of Science and Technology, Taichung 406, Taiwan, R.O.C.; 6 Medical administrative center, Show Chwan Memorial Hospital, Changhua 500, Taiwan.; 7 UPMC Hillman Cancer Center, University of Pittsburgh, Pittsburgh, PA 15260, USA.; 8 Department of Pathology, University of Pittsburgh, Pittsburgh, PA 15260, USA.; 9 Department of Biological Science and Technology, China Medical University, Taichung 406, Taiwan, R.O.C.; 10 Organ Transplantation Center, China Medical University Hospital, Taichung 404, Taiwan, R.O.C.; 11 Cell Therapy Center, China Medical University Hospital, Taichung 404, Taiwan, R.O.C.; 12 Department of Pharmacology, School of Medicine, College of Medicine, Taipei Medical University, Taipei 110, Taiwan, R.O.C.; 13 School of Post-baccalaureate Chinese Medicine, College of Chinese Medicine, China Medical University, Taichung 404, Taiwan, R.O.C.; 14 Master Program in Graduate Institute of Cancer Biology and Drug Discovery, Taipei Medical University, Taipei 110, Taiwan, R.O.C.

**Keywords:** Glioblastoma, Anthraquinone, CC12, LYN, Metastasis, Epithelial-mesenchymal transition

## Abstract

**Background:** Glioblastoma multiforme (GBM) is the most lethal malignancy in brain, which is surrounded by the blood-brain barrier (BBB), which limits the efficacy of standard treatments. Developing an effective drug that can penetrate the blood-brain barrier (BBB) remains a critical challenge in the fight against GBM. CC12 (NSC749232) is an anthraquinone tetraheterocyclic homolog with a lipophilic structure that may facilitate penetration of the brain area.

**Methods:** We used temozolomide sensitive and resistance GBM cells and animal model to identify the CC12 delivery, anti-tumor potential and its underlying mechanism.

**Results:** Importantly, toxicity triggered by CC12 was not associated with the methyl guanine-DNA methyl transferase (MGMT) methylation status which revealed a greater application potential compared to temozolomide. Alexa F488 cadaverine-labelled CC12 successfully infiltrated into the GBM sphere; in addition, ^68^Ga-labeled CC12 was also found in the orthotopic GBM area. After passing BBB, CC12 initiated both caspase-dependent intrinsic/extrinsic apoptosis pathways and apoptosis-inducing factor, EndoG-related caspase-independent apoptosis signaling in GBM. RNA sequence analysis from The Cancer Genome Atlas indicated that LYN was overexpressed in GBM is associated with poorer overall survival. We proved that targeting of LYN by CC12 may diminish GBM progression and suppress it downstream factors such as signal transduction and activator of extracellular signal-regulated kinases (ERK)/transcription 3 (STAT3)/nuclear factor (NF)-κB. CC12 was also found to participate in suppressing GBM metastasis and dysregulation of the epithelial-mesenchymal transition (EMT) through inactivation of the LYN axis.

**Conclusion:** CC12, a newly developed BBB-penetrating drug, was found to possess an anti-GBM capacity via initiating an apoptotic mechanism and disrupting LYN/ERK/STAT3/NF-κB-regulated GBM progression.

## 1. Introduction

In 2021, about 308,000 new patients were diagnosed with central nervous system (CNS) tumors, while the death toll reached 251,329 1. Intracranial glioblastoma multiforme (GBM) is the most prevalent and lethal type of brain tumor, accounting for over 70% of all cases 2. GBM is highly aggressive, characterized by high recurrence rates and poor prognoses with a 5-year survival period of about 10% 3. Currently, the standard treatment for GBM is surgery, followed by radiation and chemotherapy 4. Despite the fact that new treatment options, such as therapeutic targets, molecular therapy, gene therapy, and immunotherapy, have recently been developed, none of them has significantly increased the median survival of GBM patients.

The treatment market for GBM in 2016 was estimated to be US$465 million and it is expected to reach US$1 billion by 2025, being equally distributed between Europe, the US, Asia, and other countries 5. Although temozolomide (TMZ) is one of the major chemotherapeutic agents for GBM, serious side effects and no obvious survival benefits of patients highlight the need for new treatment options 6. Furthermore, the highly infiltrative phenotype of GBM consistently allows the growth of residual tumor cells after chemotherapy; thus, GBM clones are more aggressive and resistant to therapy and are more damaging than the primary tumor 7. One other limiting factor for chemotherapy is that the blood-brain barrier (BBB) restricts the delivery efficacy of drugs and diminishes the sensitivity to therapeutic effects 8. For instance, the efficacy of TMZ is compromised by its inability to cross the BBB and by its very short half-life (~2 h), which necessitates the use of high doses in order to achieve desired therapeutic effects 5. Despite significant advancements in cancer therapeutics, there are still numerous signaling networks and mechanisms involved in carcinogenesis, therapy resistance, and cancer recurrence that pose significant challenges in cancer treatment and management 9.

The LYN proto-oncogene, Src family tyrosine-kinase, belongs to the Src tyrosine kinase (SFK) family, the members of which are known to play roles in regulating the progression of various tumors 10,11. Kim et al. indicated that LYN can be used as a prognostic marker and a selective target of non-small cell lung cancer (NSCLC) 12. Zhang et al. also demonstrated that LYN knockdown may inactivate AKT/mammalian target of rapamycin (mTOR)-mediated human gastric cancer progression. Interestingly, the LYN expression level was found in GBM samples and was involved in promoting the migration of GBM cells 13. Activation of SFKs was also reported to affect the epithelial-mesenchymal transition (EMT) process and thus dysregulation of cytoskeletal organization and interruption of the regulation of cell-cell adhesion or adhesion to the extracellular matrix (ECM) 14.

Due to the higher efficacy and safety compared to traditional chemotherapeutic drugs, small-molecule drug candidates for treating human cancers have transformed the concept of oncological medicine 15. Our in-house synthesized multi-target small molecules have exhibited great therapeutic success with translational relevance for treating cancers 16-19. Synthetic anthraquinone analogs were discovered to possess promising anticancer, antibacterial, antiviral, and antidiabetic effects 20,21. In this study, we developed a synthesized anthraquinone tetraheterocyclic homologue, naphtho[2,3-f]quinoxaline-7,12-dione (CC12; NSC749232). Herein, we demonstrate that LYN-kinase mediated aggressive phenotypes of GBM. Consequently, we demonstrated that CC12 suppressed the proliferation and oncogenic phenotypes of GBM *in vitro* and *in vivo* GBM-bearing tumor xenograft mice via target inhibition of the Lyn signaling network. CC12 has a highly lipophilic structure that may prompt penetration across the BBB. We, therefore, suggest that CC12 might represent a new anti-GBM candidate for treating GBM.

## 2. Material and Methods

### 2.1 Cell culture of GBM8401 and T98G cells

T98G cells were provided from Taipei Medical University, Taipei, Taiwan, with Human Cell STR Profiling. GBM8401 cells a permanent human brain malignant glioma cell line provided from Tri-Service General Hospital, Taipei, Taiwan. Reagents used in this study are listed in Table 1. GBM8401 and T98G cells were respectively maintained in RPMI-1640 and Dulbecco's modified Eagle medium (DMEM), with 10% fetal bovine serum (FBS) and a 1% penicillin/streptomycin mixture. Cells were incubated in a 37 °C incubator with 5% CO_2_.

### 2.2 Cell viability assay

GBM8401 and T98G cells were seeded in 96-well plates, at a density of 7.5×10^3^ cells/well overnight and treated with 2.5, 5, 10, 15, and 20 μM CC12 and 50, 100, 150, 200 μM TMZ, respectively, for 48 h. The medium was replaced with 100 μl MTT reagent (0.5 mg/ml) for 2 h. The MTT medium was removed and replaced with 100 μl DMSO. The absorbance was detected on a Multiskan FC microplate reader (ThermoFisher Scientific, Waltham, MA, USA) at 570 nm.

### 2.3 *In vitro* BBB model (mono-co-culture and co-glia-co-culture)

In vitro monolayer BBB model's establishment was followed by the procedure in Tornabene et al. study 22. In brief, the upper channel of transwell will be covered with bEnd.3 cells (an endothelial cell from cortex) and the bottom channel was seeded with GBM8401 cells, which was known as mono-co-culture system. In addition, co-glia-co-culture system was also established and followed by Li et al. study 23. In brief, we seeded glial cells (1×10^5^ SVG-p12) on the reverse side of a 0.4 μm 24 well transwell while 1×10^5^ bEnd.3 cells (brain endothelial cells) were seeded on the other side. After 3 days the transwell was placed into a well with GL261 cells to established co-glia co-culture model. After 24-48 hr, the transendothelial electrical resistance (TEER) value between the upper and lower wells, which was greater than 150 Ωcm^2^ and 300 Ωcm^2^, respectively. The Alexa-F488-Cadaverine-labelled with 5FU (positive control), and -labelled with CC-12 were added into top well for 24-48 hr, respectively, and the transwell membrane and bottom well of chamber were fixed by 4% paraformaldehyde and stained with DAPI (for nuclear). The penetration percentage and localization pattern of Alexa-F488-Cadaverine-labeled 5FU and CC-12 on bEnd.3 cells and GBM8401 cells were identified by Confocal Microscope Detection System, Leica TCS SP2.

### 2.4 Sphere-formation and CC12 infiltration assay

GBM8401 spheres that formed in self-made 24-well plates were treated with Alexa-F488-Cadaverine-labelled CC12 for 0, 12, and 48 h. Spheres were then fixed with 4% paraformaldehyde and stained with DAPI for 15 min. The fluorescent signal from the spheres was captured by the ANDOR Dragonfly 200 High Speed Confocal System (Oxford Instruments, Abingdon, Oxfordshire, UK) and was post-processed with Imaris Microscopy Imaging Software version 9.9.

### 2.5 Xenograft, orthotopic GBM animal model and *in vivo* magnetic resonance imaging (MRI)

The animal study was approved by the IACUC of China Medical University with approval ID: CMUIACUC-2019-020. Model establishment followed a previous study 24. Six-week-old male BALB/cAnN.Cg-Foxn1nu/CrlNarl nude mice (20~25 g, *n*=5) were intracerebrally inoculated with GBM8401 (10^5^ cells) for *in vivo* MRI and GBM8401/*NF-κB-luc2* (10^5^ cells) for the *in vivo* imaging system (IVIS). Mice were divided into two groups after the tumors had reached 15 mm^3^, which included 0 and 10 mg/kg body weight CC12. Mice were kept under anesthesia (1%~2% isoflurane) and were scanned with a Brucker 7T PharmaScan scanner on days 0 and 21 using the T2-RARE sequence 25. Tumor volumes and surface areas on T2-RARE images were quantified by customized MATLAB scripts (The Mathworks, Natick, MA, USA). GBM8401 shLYN and GBM8401 OE-LYN cells were inoculated into the right flank (10^7^ cells) of mice and allowed to progress.

### 2.6 Molecular docking analysis

The three-dimensional (3D) crystal structure of the LYN-kinase (PDB:1R21) was retrived in PDF format from the protein data bank and converted to PDBQT format using AutoDock Vina software (vers. 0.8) 26. The compound (CC12) was built using the Avogadro tool 27. The compound and the protein were subjected to a pre-docking simulation according to methods described in previous studies 28,29. Docking was conducted using default settings of AutoDock Vina as described in a previous study 30. Docking results were visualized and analyzed using the PyMOL algorithm, Discovery studio visualizer 31, and the PLIP server.

### 2.7 Statistical analysis

To ensure the robustness and reproducibility of our results, all experiments were repeated at least three times. For statistical analysis, we utilized Graphpad Prism 7.0 software and employed a one-way analysis of variance (ANOVA) with post-hoc Tukey's Honestly Significant Difference Test for comparing more than one group, and the student t-test (parametric) method for pair-wise comparisons. If the statistical value between groups was smaller than 0.05, it was defined as a significant difference. All of the values in this work are presented as the mean ± standard error (SE).

The details of materials and other methods are described in the Supplementary information.

## 3. Results

### 3.1 CC12 is a potential BBB-permeating molecule for GBM

We conducted a preliminary in silico evaluation of the blood-brain barrier (BBB) permeation ability of CC12, using ALzPlatform 32, a BBB prediction algorithm. The model was built on training sets of four classes of fingerprints consisting of 1593 compounds by applying the support vector machine (SVM) and LiCABEDS algorithms. Interesting, our results revealed the potential drug-likeness of CC12 with the ability to cross the BBB.

**Figure 1:**
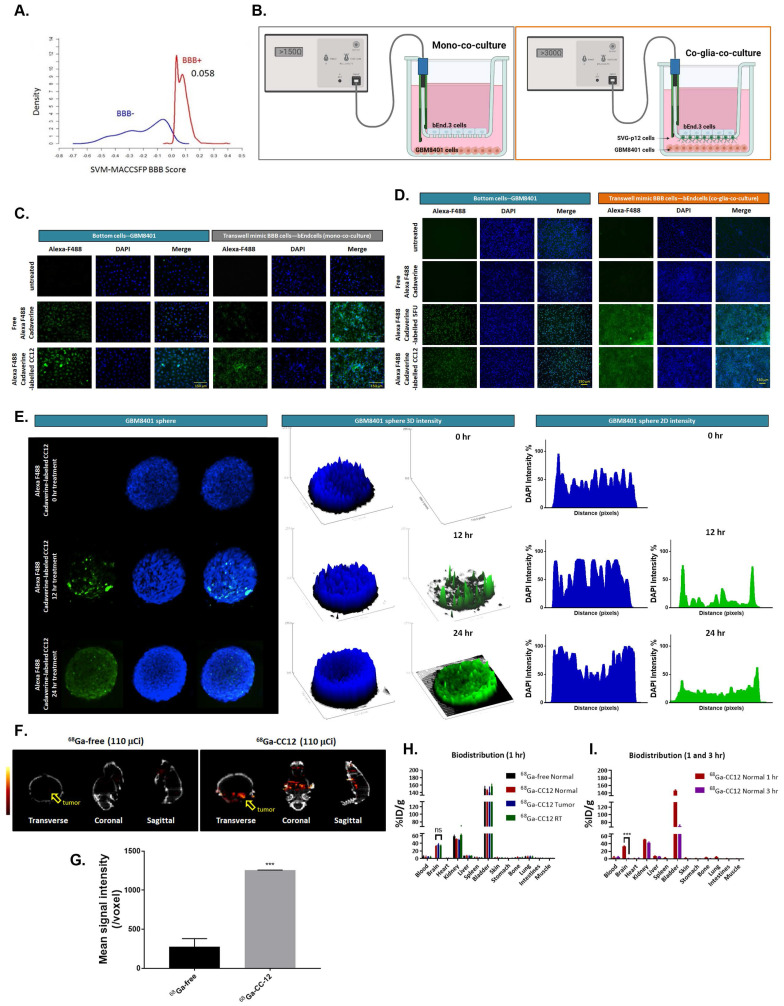
** CC12 is a potential blood brain barrier (BBB)-permeant molecule for targeting LYN-kinase.** (A) In silico BBB permeability curve of CC12 based on support vector machine (SVM) and LiCABEDS algorithms is displayed. (B) The schematic diagram of two different *in vitro* BBB models. The penetration and infiltration pattern of Alexa-F488-Cadaverine-labelled CC12 assayed by (C-D) *in vitro* BBB model and (E) GBM8401 sphere are displayed. (F) SPECT scan is acquired after injection of 110 μCi ^68^Ga-CC12, and ^68^Ga-free. (G) The mean intensity of each group and signal intensity of ^68^Ga-CC12 and ^68^Ga-free are displayed. (H) Bio-distribution of ^68^Ga-CC12 on normal mice, brain RT exposure mice, and brain tumor bearing mice organs are collected and detected by γ-counter after one hours' injection. (I) Bio-distribution of ^68^Ga-CC12 on normal mice after 1 hr and 3 hr injection of ^68^Ga-CC12. (ns= no significant differences; ^***^p<0.005 vs. ^68^Ga-free or normal 3 hr)

CC12 achieved the BBB permeability score of 0.058 on a cut of scale of 0.02 (Figure 1A, S1A). This is comparable to the BBB permeability value of 0.077 obtained for 5-Fluorouracil (S2A) a well-known BBB-penetrating agent, while temozolomide a poor BBB penetrating agent achieve a low BBB-permeability score of 0.029 (S2B). In addition, the drug demonstrated non-substrability and non-inhibitor tendencies towards P-glycoprotein (P-gp) as modeled in Figure S1B. Additionally, we developed *in vitro* BBB model to investigate whether CC12 may truly pass through BBB. Figure 1B, we demonstrated two type of *in vitro* BBB models as described in material section, including mono-co-culture and co-glia co-culture (bEnd3/SVG-p12/GBM8401). Figure 1C-D illustrated the detection of Alexa F488 cadaverine-labeled CC12 on the bottom wells of both GBM8401 cells and BBB mimic brain-derived endothelial cells, bEnd3 or bEnd3/SVG-p12 co-culture cells attached membranes. In addition, a positive control using Alexa F488 cadaverine-labeled 5-FU was employed to confirm the drug's ability to penetrate the BBB (Figure 1D). Then, we further investigated the infiltration capacity of CC12 in GBM8401 spheres. As indicated in Figure 1E, Alexa F488 cadaverine-labeled CC12 had successfully infiltrated into GBM8401 spheres after 24 h of treatment. To determine the distribution of Alexa F488 cadaverine-labeled CC12 in spheres, we employed both 2D and 3D analysis models. The results of both models revealed an even distribution of the drug throughout GBM8401 cells after 24 hours of treatment. Next, to demonstrate the *in vivo* delivery potential of CC12 within the GBM area, we labeled CC12 with ^68^Ga and evaluated the accumulation of ^68^Ga-CC12 and ^68^Ga-free *in vivo* by Nano-SPECT/CT scan immediately (<10 min) after intravenous injection (Figure 1F). The CC12 label efficacy of ^68^Ga was represented as 82.78% (Figure S3). The mean signaling intensity per voxel in the region of interest was 4.4 times higher for ^68^Ga-CC12 than for the free form of ^68^Ga (Figure 1G). This significant difference suggests that CC12 has the ability to penetrate the BBB and accumulate in the brain area (Table 3). Moreover, we conducted experiments to allow for longer drug distribution time (1hr) and investigate the concentration difference of CC12 between normal, tumor, and radiation-exposed (6 Gy whole-brain) brain regions. Figure 1H illustrated that there was no significant difference in ^68^Ga-CC12 concentration between the three types of models after 1 hour of injection, indicating that the ^68^Ga-CC12 had successfully infiltrated the brain area in all models as compared to ^68^Ga-free (Table 4). Additionally, ^68^Ga-CC12 was found to be washed out of normal brain tissue within 3 hours (Figure 1I and Table 5). Due to vessel abnormality, the accumulation rate of CC12 in tumor tissue was faster than that in normal tissue, resulting in a %ID/g that was 90 times higher in the tumor area than in the normal brain area immediately after injection. Ultimately, %ID/g value in brain area reached a similar level after 1 hour in normal, tumor, and radiation-exposed model. In conclusion, our study suggested that CC12 is a potential BBB-permeant molecular drug**.**

### 3.2 CC12 induced cytotoxicity and apoptosis of GBM independent of the methyl guanine methyl transferase (MGMT) methylation status

**Figure 2:**
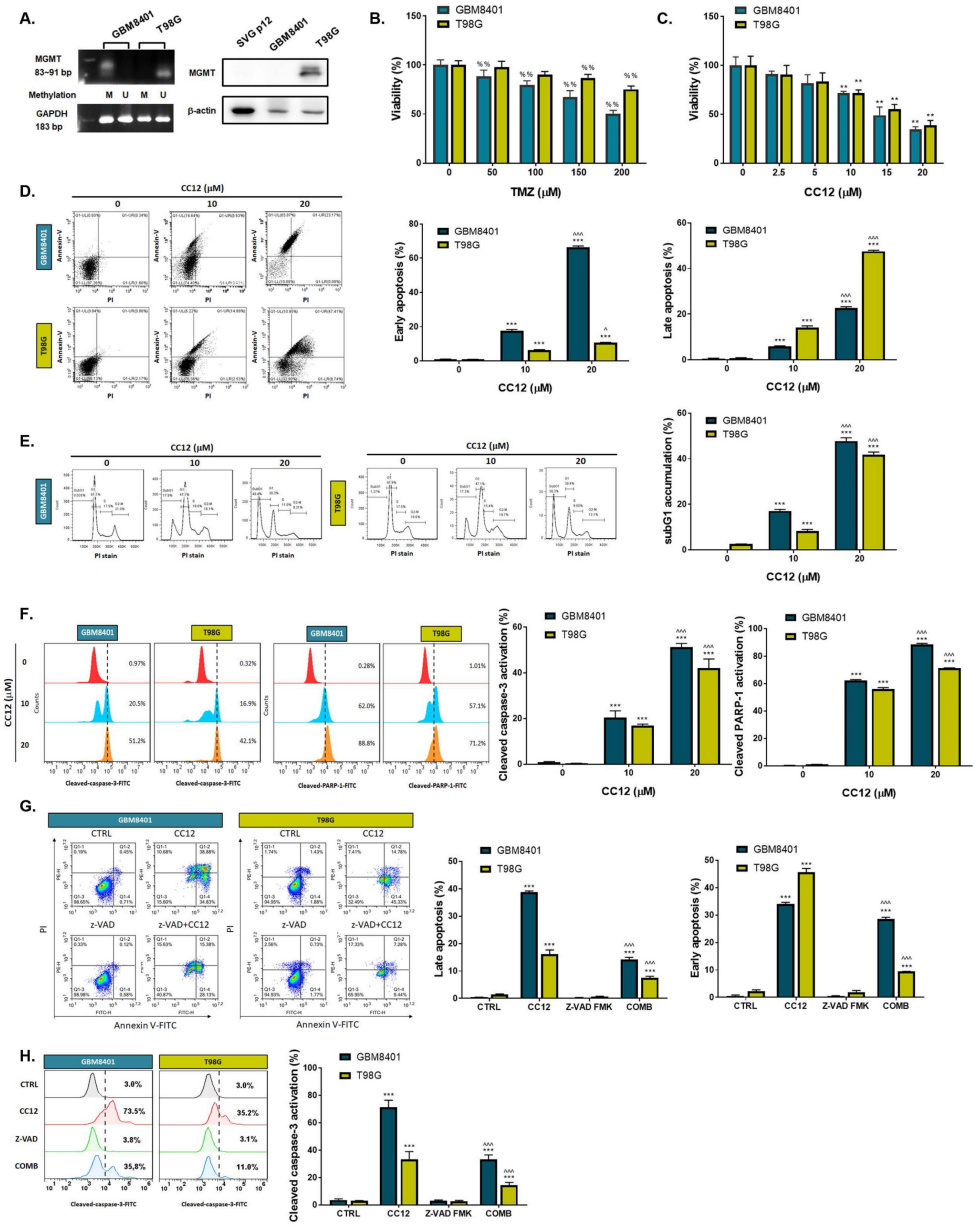
** CC12 induced apoptosis is not associated with MGMT expression.** (A) The methylation status of GBM8401 and T98G cells is assayed by methylation-specific PCR and Western blotting. SVG-p12 was represented as normal glia cells. (U: unmethylation; M: methylation). (B-C) The cytotoxicity effect of TMZ and CC12 in GBM cells are performed by MTT assay. The staining of (D) Annexin-V/propidium iodide (PI) and (E) cells cycle are performed by flow cytometry. (F) The staining of cleaved caspase-3 and cleaved poly (ADP ribose) polymerase (PARP)-1 are detected by flow cytometry. The staining of (G) annexin-V/PI and (H) cleaved caspase-3 in CC12 combined with Z-VAD FMK (pan-caspase inhibitor) are validated by flow cytometry. (^%%^*p*<0.01 vs. 0 µM TMZ; **p<0.01, ***p<0.005 vs. 0 µM CC12; ^*p*<0.05, ^^^*p*<0.005 vs. 10 µM CC12)

**Figure 3:**
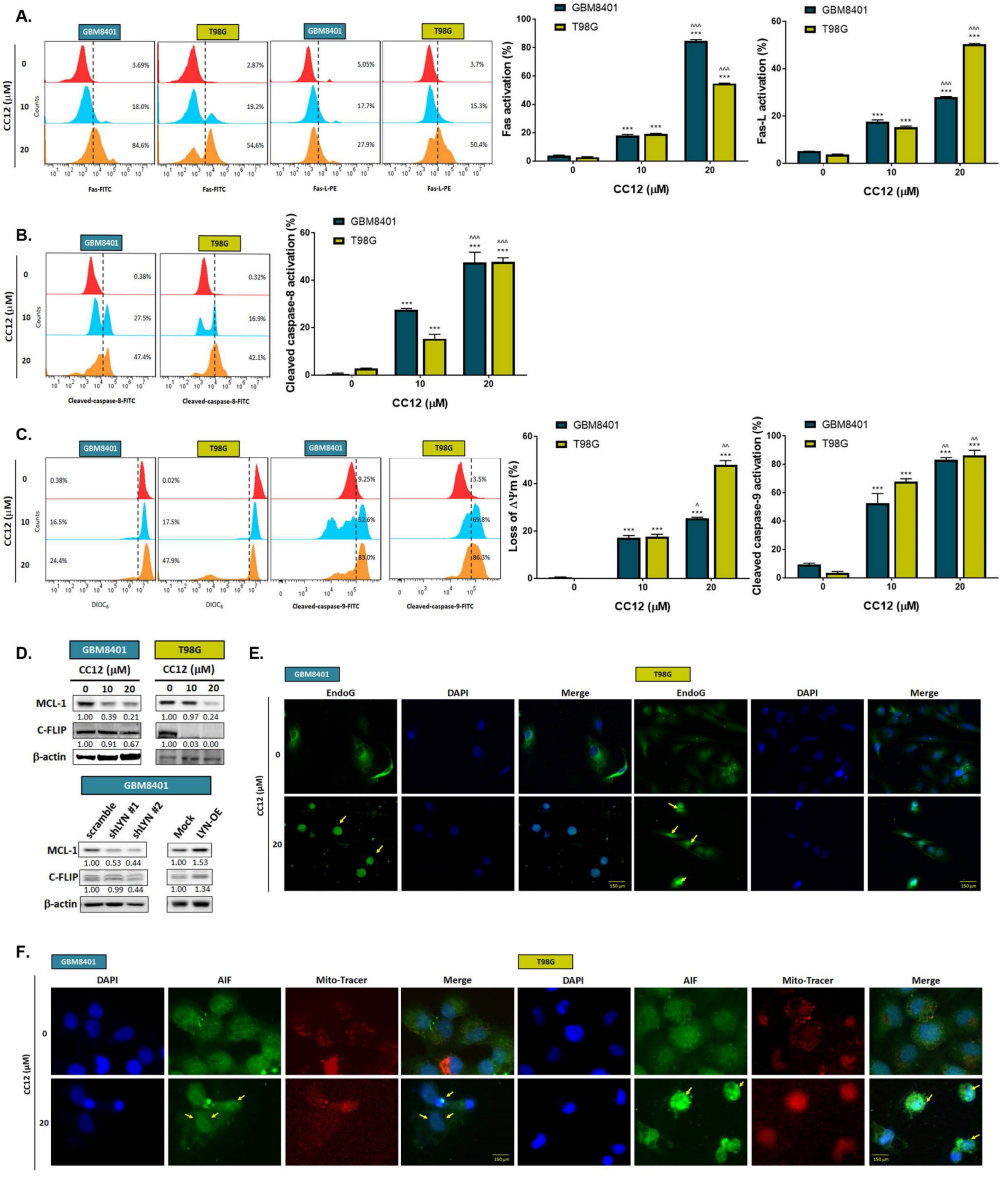
**Caspase-dependent and -independent apoptosis pathways are activated by CC12.** Flow cytometric staining of (A) Fas, Fas-L, (B) cleaved caspase-8, (C) DIOC_6_, and cleaved caspase-9 as detected by flow cytometry. (D) Western blot analysis showing protein expressions of MCL-1 and C-FLIP after CC12, shLYN, and LYN-OE treatment. (E-F) The nuclear translocation of EndoG and apoptosis-inducible factor (AIF) was determined by IF staining. (**p<0.01, ***p<0.005 vs. 0 µM CC12; ^*p*<0.05, ^^^*p*<0.005 vs. 10 µM CC12).

The methylation status of the cell lines was validated by a methylation-specific polymerase chain reaction (PCR) and Western blotting which revealed an MGMT methylation status of GBM8401 cells and an unmethylated status of T98G cells (Figure 2A). T98G cells were found to be relatively less sensitive to TMZ standard treatment than were GBM8401 cells (Figure 2B). Interestingly, we found that CC12 induced dose-dependent cytotoxic effects against both GBM8401 and T98G cells (Figure 2C). In addition, both early (Annexin-V^+^/PI^-^) and late apoptosis (Annexin-V^+^/PI^+^) were significantly induced by CC12 in the two GBM cell lines (Figure 2D). CC12 may also increase the accumulation of the subG_1_ phase population which represented apoptotic cell death (Figure 2E). We further identified the caspase-dependent apoptosis role of CC12 in both GBM8401 and T98G cells. As shown in Figure 2F left panel, activation of cleaved caspase-3 was induced by CC12. Furthermore, poly(ADP ribose) polymerase (PARP)-1 that may be cleaved by caspase-3 33 was also found to be initiated after CC12 treatment (Figure 2F right panel). We also confirmed that the apoptosis induction effect from CC12 is associated with caspase-dependent signaling by combining with caspase-inhibitor Z-VAD (Figure 2G-H). The activation of cleaved caspase-3 and annexin-V were markedly decreased in Z-VAD plus CC12 group, while part of apoptosis may still remain. These results imply CC12 induce GBM cells death may not only mediate by caspase-dependent signaling. To further identify whether both death receptor-dependent apoptosis (through an extrinsic apoptosis pathway) and mitochondrion-dependent apoptosis (through an intrinsic apoptosis pathway) were both activated by CC12, we performed validation by flow cytometry. Figure 3A-3B show that the extrinsic apoptosis pathway, including Fas, Fas-L, and cleaved caspase-8, was markedly activated by CC12 in GBM8401 and T98G cells. On the other hand, loss of ΔΨm and cleaved caspase-9 was promoted by CC12 (Figure 3C). Additionally, anti-apoptosis-related genes including MCL-1 and C-FLIP were both found to be suppressed by CC12 and shLYN (Figure 3D). Not only was caspase-dependent apoptosis induced, the nuclear translocation of EndoG and apoptosis-inducing factor (AIF) which mediated caspase-independent apoptosis was triggered by CC12 as well (Figure 3E-F). In summary, CC12-induced cytotoxicity of GBM cells was associated with induction of both caspase-dependent and -independent apoptosis pathways.

### 3.3 CC12-mediated anti-GBM toxicity is associated with LYN inactivation

**Figure 4:**
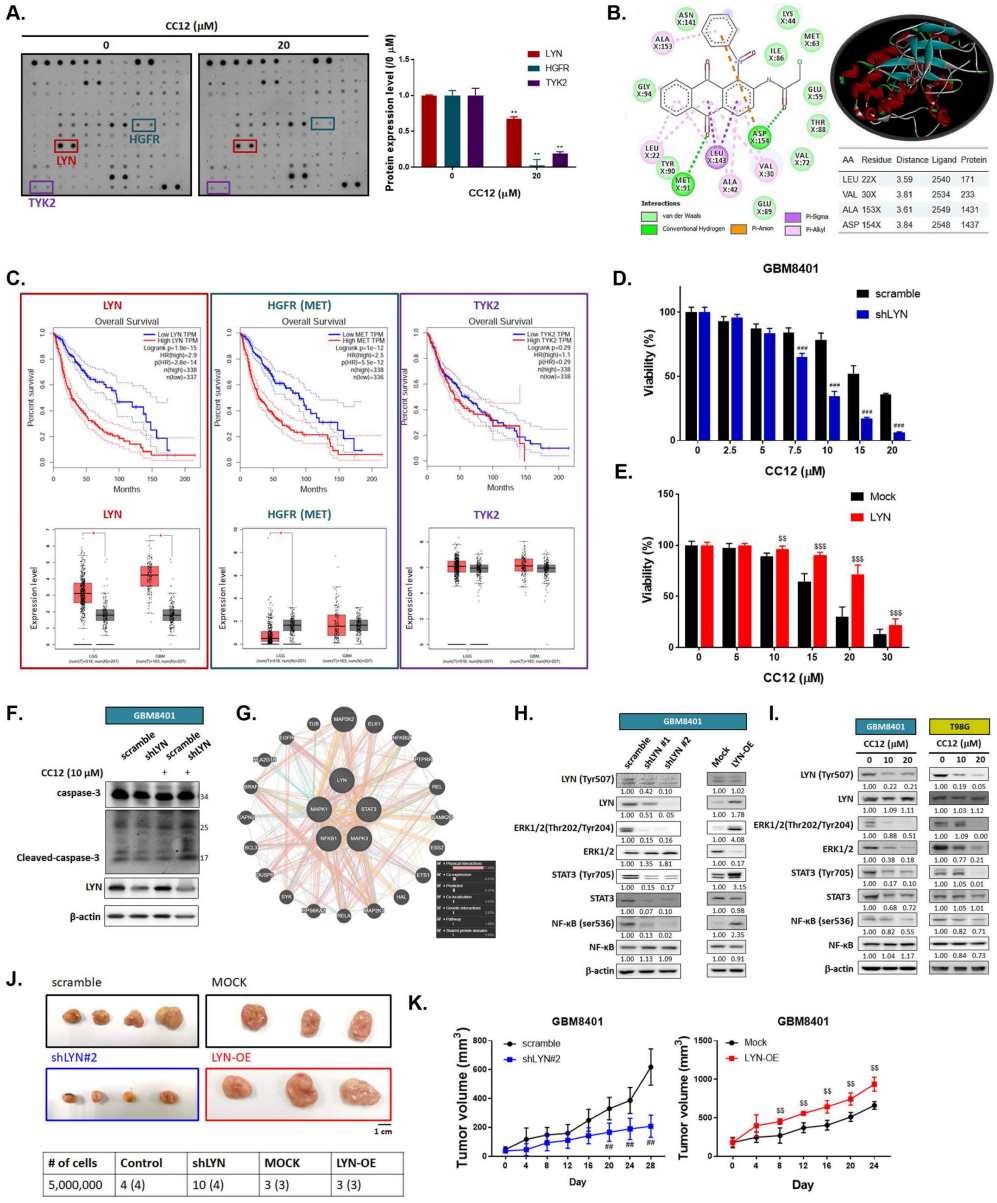
** LYN inactivation may enhance the anti-GBM potential of CC12.** (A) A human phospho-kinase antibody array was performed on GBM8401 cells after CC12 treatment. (B) Two-Dimensional (Left panel) and three-dimensional (right upper panel) view of the CC12 molecules interacting with the LYN amino acid at the binding site. (C) The survival pattern depends on LYN, HGFR and TKY2 expression level and expression level in LGG and GBM sample are performed by GEPIA (http://gepia.cancer-pku.cn/). Cell viability analysis of CC12 on (D) shLYN GBM8401 and (E) LYN-OE GBM8401 cells as performed by an MTT assay. (F) Cleaved caspase-3 expression level of in shLYN GBM8401 and LYN-OE GBM8401 cells after treated with CC12 was assayed by Western blotting. (G) The correlation of LYN, signal transduction and activator of transcription 3 (STAT3), extracellular signal-regulated kinase (ERK), and nuclear factor (NF)-κB genes that acquired from GeneMANIA analysis is displayed. (H-I) Western blot analysis showing the effects of shLYN, LYN-OE, and CC12 treatment on expression levels of LYN, STAT3, ERK, NF-κB. (J) Tumor-forming capacity and (K) tumor-growth pattern of silencing and overexpression of LYN are displayed. (**p<0.01 vs. 0 µM CC12; ^##^*p*<0.01, ^###^*p*<0.005 vs. scrambled; ^$$^*p*<0.01, ^$$$^*p*<0.005 vs. Mock).

To elucidate the molecular mechanism of CC12 in GBM cells, we utilized a human phospho-kinase antibody array. In Figure 4A, the marked inhibition of LYN, hepatocyte growth factor receptor (HGFR), and TYK2 was found after CC12 treatment. Our molecular docking analysis also revealed that CC12 could bind to LYN-kinase with a strong binding affinity (ΔG) of -9.9 kcal/mol (Figure 4B). Although the docking simulation has a limitation in its inability to detect covalent interactions, this model revealed the non-covalent (reversable) interactions occurring between CCL2 and LYN. CCL2 bound to LYN-kinase by two hydrogen bonds with the ASP154 and MET91 residues of the LYN-kinase-binding domain. The complex also formed p-pi stacking (Phe16 and Phe214), pi-anion with ASP154, and pi-sigma with the LEU143 residue. Furthermore, several alkyl interactions with ALA153, LEU22, ALA42, and VAL30 were found within the CC12_LYN-kinase complex. The high numbers of pi interactions may be attributed to the strong binding efficacy of CC12 towards LYN-kinase. In addition, the RXn-02_IDO1 complex was stabilized by a total of 10 van der Waals forces (ASN141, LYS44, ILE86, GLU59, MET63, THR88, VAL72, GLU89, TYR90, and GLY94) and several hydrophobic contacts (Figure 4B).

Next, we used GEPIA website to calculate the correlation between these genes' expressions and the percent of survival on glioma patients. As indicated in figure 4C, patient with higher expression level of LYN and HGFR may show poorer survival pattern. Additionally, LYN expression is relatively higher in both low-grade glioma (LGG) and glioblastoma (GBM). The HGFR expression level was only found to be higher in LGG patients as compared to normal tissue, but not in GBM patients. The survival and expression level have no obvious correlation in glioma patient with or without TYK2 expression level. Thus, we performed silencing and overexpression of LYN on GBM8401 cells to illustrate the role of it on GBM. In Figure 4D, cell viability was markedly decreased by CC12 in shLYN as compared to wild type GBM8401 cells. On the contrary, the induction of LYN may diminish CC12-induced cytotoxicity in GBM8401 cells (Figure 4E). The cleaved caspase-3 expression also confirmed the apoptosis effect that induced by CC12 is mediated by LYN expression (Figure 4F). Analysis from GeneMANIA indicated the interaction of LYN with MAPK/ STAT3/NF-κB signaling (Figure 4G). Silencing or overexpression LYN may not only affect the phosphorylation of LYN but also regulating the phosphorylation downstream oncogenes, such as ERK, STAT3 and NF-κB in both GBM8401 and T98G cells (Figure 4H-I). The tumor-forming capacity and growth size were reduced after silencing LYN; however, they were enhanced by LYN overexpression (Figure 4J-K). Taken together, the CC12-induced anti-GBM effect was associated with inactivation of LYN/ ERK/STAT3/NF-κB signaling.

### 3.4 CC12 suppressed the EMT-mediated invasion and migration abilities of GBM cells

**Figure 5:**
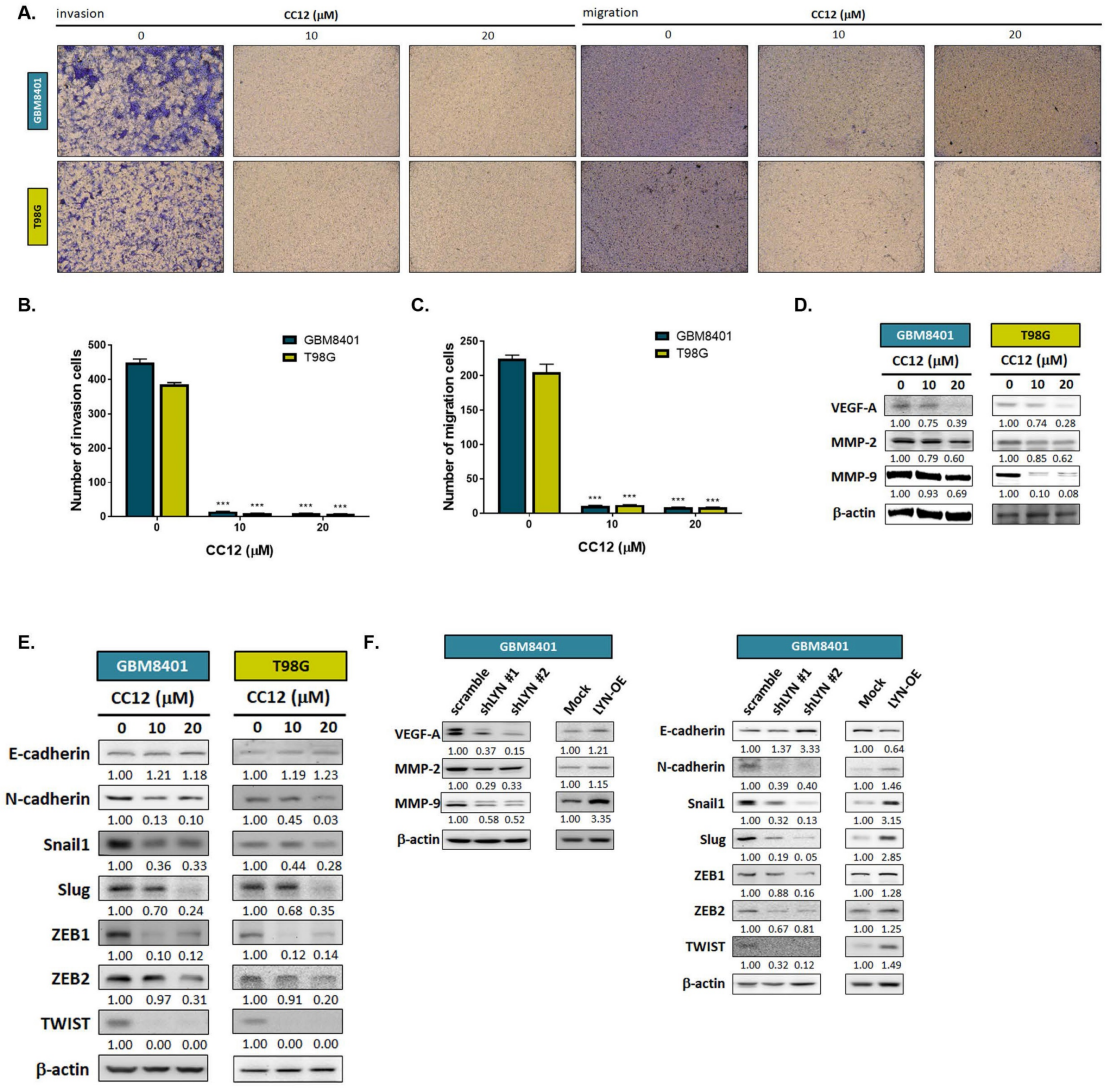
** Epithelial-mesenchymal transition (EMT)-mediated invasion and migration of GBM cells are suppressed by CC12.** (A) Invasion and migration pattern and (B-C) the quantification bar chart after CC12 treated are performed by transwell assay. (D-E) The protein expression of metastasis associated genes and EMT related genes after CC12 treatment on GBM8401 and T98G cells are assayed by Western blotting. (F) The protein expression of metastasis associated genes and EMT related genes on shLYN and LYN-OE GBM8401 cells are assayed by Western blotting. (***p<0.005 vs. 0 µM CC12)

To further investigate whether CC12 might play a role in altering the EMT and metastatic ability of GBM, we performed a transwell invasion and migration assay. As shown in Figure 5A-C, inhibition of invasion and migration was found in the CC12-treated group. In addition, the silencing of LYN in GBM8401 cells may also reduce the invasion ability of GBM8401 cells; nevertheless, overexpression LYN may trigger the invasion ability of GBM8401 cells (Figure S4). Next, we suggested that CC12 may effectively suppress metastasis-associated gene expressions in dose-dependent manners, including vascular endothelial growth factor-A (VEGF-A), matrix metalloproteinase MMP-2, and MMP-9 (Figure 5D). EMT signaling activation is a key mechanism that contributes to the metastatic process in many cancer types 34. Thus, we identified whether CC12 revocation/inhibition of the invasion and migration abilities of GBM was correlated with control of the inactivation of EMT signaling. In Figure 5E, the expression level of E-cadherin (an endothelial-specific adhesion molecule) was markedly increased in the CC12-treated group, whereas the N-cadherin expression level had decreased. Several important factors that participate in promoting the EMT process were all decreased by CC12, such as Snail1, Slug, ZEB1, ZEB2, and TWIST (Figure 5E). In the meantime, the silencing of LYN also demonstrated an inhibitory capacity against these EMT-promoting genes (Figure 5F). In contrast, overexpression of LYN may have initiated N-cadherin and EMT promotion-related gene expressions, but suppressed E-cadherin expression (Figure 5F). In summary, the CC12-suppressed migratory and invasion abilities were associated with the downregulation of LYN.

### 3.5 CC12 showed an anti-GBM capacity in an orthotopic glioblastoma animal model

**Figure 6:**
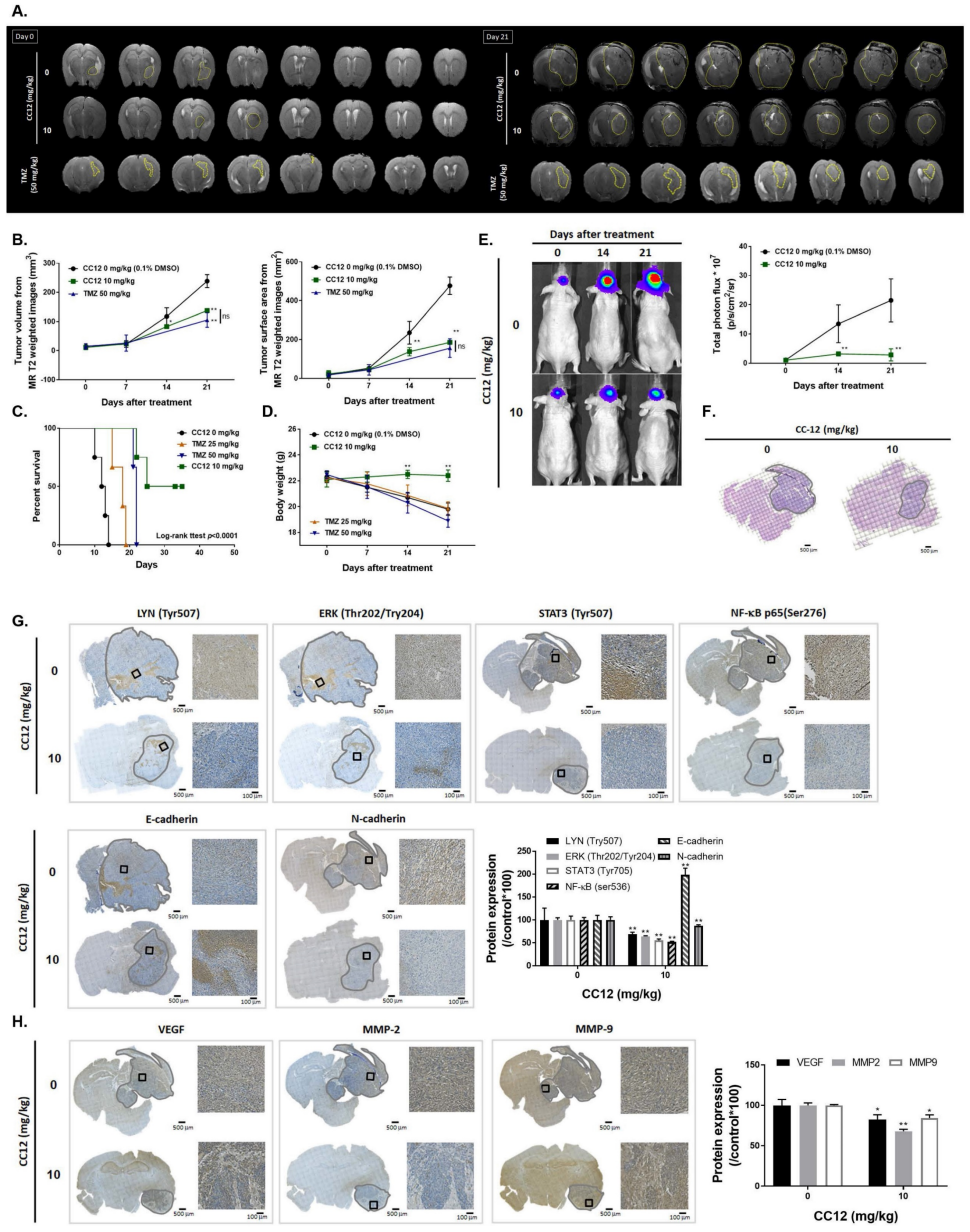

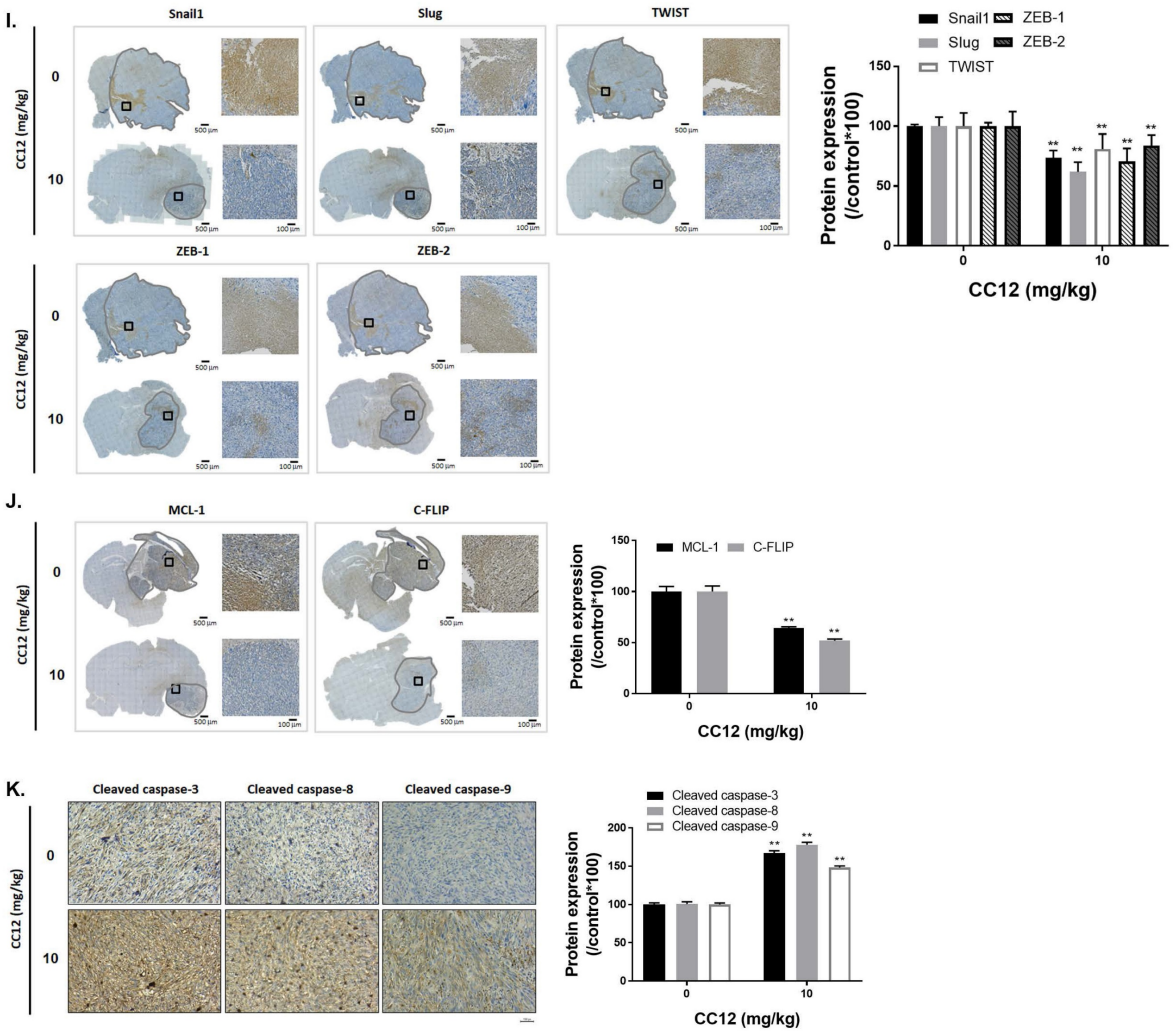
** Anti-GBM capacity of CC12 was validated by an orthotopic GBM animal model.** (A) T2-RARE images of control, CC12-treated and TMZ-treated (positive control) mice are presented. (B) Tumor volumes and tumor surface areas were calculated from T2-RARE images. The (C) survival outcome and (D) the body weight of mice is recorded. (E) Bioluminescence images of a GBM8401/*NF-κB-luc2*-bearing model after CC12 treatment on days 0, 14, and 21 are presented. (F) Tumor H&E staining on day 21 is presented HC-stained images and quantification results of (G) LYN Try507, extracellular signal-regulated kinase (ERK) Thr202/Try204, signal transduction and activator of transcription 3 (STAT3) Tyr705, nuclear factor (NF-κB) Ser536, E-cadherin, N-cadherin, (H) vascular endothelial growth factor (VEGF), matrix metalloproteinase (MMP)-2, and MMP-9, (I) Snail1, Slug, TWIST, ZEB-1, ZEB-2, (J) MCL-1, C-FLIP, (K) cleaved caspase-3, -8 and -9 are displayed. (*p<0.05, **p<0.01 vs. 0 mg/kg CC12)

To confirm CC12's cytotoxicity in our *in vitro* model, evaluate its ability to penetrate the blood-brain barrier (BBB), and establish an orthotopic GBM animal model, we conducted MR T2-RARE imaging on days 0 and 21. The imaging results demonstrated a significant suppression in GBM progression in the CC12-treated group, indicating the potential efficacy of CC12 in treating GBM (Figure 6A). Tumor volumes calculated by MR T2-RARE scans showed significant tumor inhibition potential after 7 days of treatment with maintenance of an efficient tumor control rate (Figure 6B).

**Figure 7:**
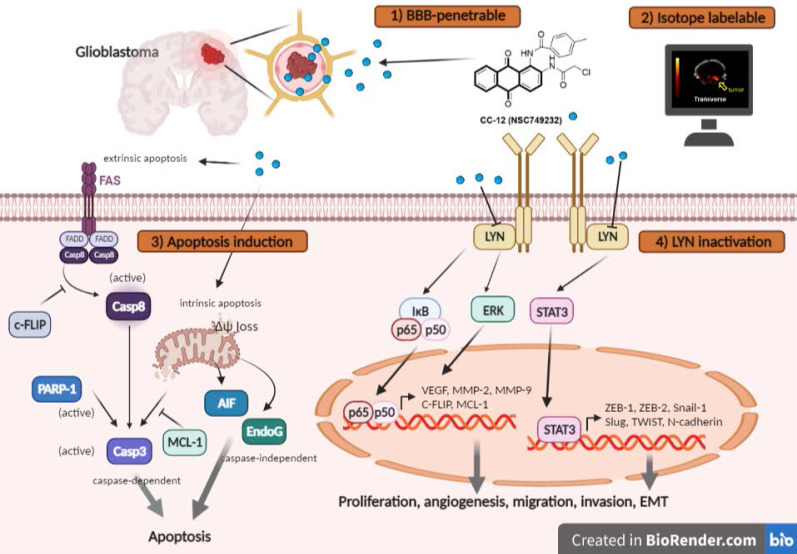
** The proposed mechanism of CC12 on GBM.** The anthraquinone tetraheterocyclic homolog, CC12, possesses a blood-brain barrier penetration ability and thus initiated an apoptotic effect in a GBM model. CC12 may induce both caspase-dependent intrinsic and extrinsic apoptosis and apoptosis-inducible factor (AIF)-mediated caspase-independent apoptosis pathways. Additionally, CC12 may inactivate LYN/signal transduction and activator of transcription 3 (STAT3)/nuclear factor (NF)-κB-regulated anti-apoptosis, metastasis, and the epithelial-mesenchymal transition in a GBM model. (Figure was created in Biorender.com)

Tumor surface area calculated by the MR T2-RARE scans showed a significant tumor inhibition potential after 7 days of treatment, and an efficient tumor control rate was maintained (Figure 6B). Furthermore, the tumor surface area, which was used to represent tumor progression, also decreased with CC12 treatment (Figure 6B). The inhibition efficacy has no obvious difference as compared to standard treatment TMZ. The superior survival of tumor bearing mice was showed in CC12 treated mice as compared to TMZ (Figure 6C). The reason that causes mice death in TMZ group is not only associated with tumor progression but also the toxicity of drug itself as indicated in Figure 6D mice body weight. To further identify the activation of NF-κB, we also established a GBM8401/*NF-κB-luc2*-bearing model. As illustrated in Figure 6E, *in vivo* activation of NF-κB was effectively reduced by CC12. The growth of tumor was also validated by H&E that illustrated the markedly tumor inhibition in CC12 treated group (Figure 6F). Additionally, Figure 6F shows the tumor pattern in the untreated and CC12-treated groups which corresponded to IHC-stained sections. The expression level of LYN-mediated signaling and its downstream compounds, such as ERK, STAT3, and NF-κB had all decreased by 30%~40% after CC12 treatment (Figure 6G). Corresponding to the *in vitro* results, CC12 may have suppressed expression of the N-cadherin protein but triggered E-cadherin expression, which resulted in blockage of the EMT process of GBM (Figure 6G). Consequently, metastasis- (Figure 6H) and EMT progression-related molecules (Figure 6I) all decreased due to CC12 in GBM tissues. Furthermore, anti-apoptosis proteins, including MCL-1 and C-FLIP, were suppressed by CC12 (Figure 6J), but apoptosis factors were promoted by CC12 (Figure 6K). In summary, CC12 showed potential to reach the brain area and then executed its anti-GBM capacity by downregulating LYN-mediated tumor progression and initiating apoptosis pathways (Figure 7).

## 4. Discussion

The unmethylated MGMT promoter was detected in 50% of gliomas and is known to cause cells to be insensitive to alkylating chemotherapy. Unmethylated tumors were also found to progress twice as fast as methylated tumors after radiotherapy 35. MGMT methylation may downregulate or eliminates MGMT expression, resulting in reduced DNA repair and retention of alkyl groups, thereby facilitating greater efficacy of alkylating agents such as TMZ 36. Thus, the MGMT methylation status is recognized as a reliable predicator of susceptibility to adjuvant therapy and prognosis of GBM. In this study, we indicated that the CC12 treatment efficacy was not affected by the MGMT status, which implied the wide application potential of CC12 against GBM. Both GBM8401 (with methylated MGMT) and T98G (with unmethylated MGMT) were sensitive to CC12 treatment compared to TMZ treatment (Figure 2B-C). Furthermore, deregulation of apoptotic cell death was found in GBM and was associated with activation of NF-κB 37. The cellular FLICE-inhibitory protein (C-FLIP) is one of the downstream signal molecules/genes from NF-κB that disrupts activation of apoptosis by interacting with caspase-8 38. Downregulating myeloid cell leukemia (MCL)-1, another NF-κB downstream gene, showed potential to enhance the induction of apoptosis in GBM 39. In Figure 3D, expressions of C-FLIP and MCL-1 were markedly decreased by CC12, and caspase-dependent apoptosis had increased with CC12. Moreover, activation and phosphorylation of NF-κB were effectively suppressed by CC12. The nuclear translocation of apoptosis-inducing factor (AIF) from mitochondria to nuclei may be associated with activation of apoptosis and was correlated with STAT3 inhibition 40. CC12 also suppressed STAT3 phosphorylation, and the nuclear translocation of AIF resulted in apoptosis activation in GBM (Figure 3E-F and 4I). In summary, CC12-mediated toxicity in GBM is associated with caspase-dependent and caspase-independent apoptosis pathways and might not be restricted by the methylation status of MGMT.

40%~50% of GBM were found to possess epidermal growth factor receptor (EGFR) gene amplifications and/or mutation characteristics 41. Targeting LYN, STAT3, and NF-κB showed potential for inhibiting cell motility and tumor growth in EGFRvIII-expressing head and neck cancers 42,43. Additionally, LYN-kinase and other Src kinase families were proven to be involved in activation of STAT3-mediated and NF-κB-mediated tumor progression 44. We demonstrated that patients with higher LYN expression possessed poor overall survival and had greater risks of progression into a more-aggressive type of glioma (Figure 4C). CC12 showed potential to decrease the protein kinase activity of LYN in GBM cells (Figure 4A) and showed superior toxicity in LYN-knockdown GBM cells (Figure 4D). We demonstrated that the phosphorylation of LYN, STAT3, and NF-κB was effectively diminished by CC12 treatment (Figure 4I). CC12-induced GBM toxicity was associated with inactivation of the LYN/STAT3/NF-κB axis.

Moreover, depletion of LYN markedly attenuated EGFRvIII-induced signaling transduction and thus suppressed the migration of GBM cells 45. Our migration and invasion results indicated that CC12 might not only suppress the invasion and migratory abilities of GBM but also reduced expressions of metastasis associated genes, such as VEGF, MMP-2, and MMP-9. Activation of LYN is also known to participle in promoting the EMT process in various types of cancers 46,47. ZEB homeobox is a transcription factor family that includes ZEB1 and ZEB2, which may induce the EMT during cancer development 48. The binding of EMT transcription factors, including Snail, Slug, and TWIST, to the E-cadherin promoter may lead to repression of E-cadherin, thus initiating the EMT process 49. In contrast, the mesenchymal transition gains function by the upregulation of N-cadherin 50. Our data indicated that EMT promotion-related genes, such as ZEB-1, ZEB-2, Snail1, Slug, TWIST, and N-cadherin, were all suppressed by CC12 (Figure 5E and 6G-I). On the contrary, induction of E-cadherin was observed in CC12-treated cells and tumor tissues (Figure 5E and 6G). LYN-knockdown significantly suppressed the metastatic capacity, attenuated the development of the EMT, and inhibited expressions of genes related to these processes. In conclusion, CC12 effectively suppressed the ability of invasion/migration and EMT development (Figure 5) and their associated protein expressions in a GBM model via inactivating LYN-mediated signaling transduction.

Molecular docking is a widely explored simulation tool for modeling the potential binding efficacy of a small-molecule drug candidate and its targeted protein molecules, and for evaluating interactions with the protein-ligand docked complex 16,51, thus providing insights into the biological activities of the drug candidate and aiding drug discovery and development 52,53. Consequently, our molecular docking analysis revealed that CC12 bound to LYN-kinase with a strong binding affinity (ΔG) of -9.9 kcal/mol (Figure 2B). CCL2 bound to LYN-kinase by two hydrogen bonds with the ASP154 and MET91 residues of the LYN-kinase-binding domain. The complex also formed p-pi stacking (Phe16 and Phe214), pi-anion with ASP154 and pi-sigma with the LEU143 residue. Furthermore, several alkyl interactions with ALA153, LEU22, ALA42, and VAL30 were found within the CC12_LYN-kinase complex. The high numbers of pi interactions may be attributed to the strong binding efficacy of CC12 towards LYN-kinase. In addition, the RXn-02_IDO1 complex was stabilized by a total of 10 van der Waals forces (ASN141, LYS44, ILE86, GLU59, MET63, THR88, VAL72, GLU89, TYR90, and GLY94) and several hydrophobic contacts.

To evaluate the penetration of CC-12, we used support vector machine (SVM) and LiCABEDS algorithms, as shown in Figure 1A and S2, and compared it to 5-FU and TMZ. We also used *in vitro* BBB model to proof the infiltration potential of CC-12 as compared to 5-FU (positive control) (Figure 1D). In our bio-D assay (Figure 1F-I), we found that CC-12 accumulated faster in mice with tumors (<10 min), while a signal was also observed in normal brain at a later time point (1 hr), indicating its potential to penetrate the blood-brain barrier. In addition to the permeability of the drug itself, the hyperpermeability of tumor vessels in the GBM model also need to be consider. This can be attributed to the overexpression of vascular endothelial growth factor/vascular permeability factor (VEGF/VPF) in tumor blood vessels, which may enhance the penetration rate of the drug compared to normal blood vessels 54.

These studies not only indicated the anti-GBM potential of our self-synthesized BBB-penetrable drug, CC12, but also successfully presented the mechanism of its action. CC12 efficiently initiated caspase-dependent and caspase-independent apoptosis pathways and inactivated LYN-mediated metastasis and EMT development in GBM. CC12 is recognized to act as a potential anti-GBM drug by targeting apoptosis and metastasis mechanisms in GBM.

## Supplementary Material

Supplementary methods and figures.

## Figures and Tables

**Table 1:** Reagents that used in this study.

Reagents	Company	Cat no. or product no.
RPMI-1640	Thermo Fisher Scientific, Fremont, CA, USA	11875176
DMEM	Thermo Fisher Scientific	11965084
Fetal Bovine Sera	Hyclone Laboratories, Inc, Utah, UK	SH30396.02HI
Penicillin/streptomycin	Thermo Fisher Scientific	15140122
DMSO	Sigma-Aldrich, St.Louis, MO, USA	CAS 67-68-5
1-(4,5-Dimethylthiazol-2-yl)-3,5-diphenylformazan, MTT	Sigma-Aldrich	CAS 57360-69-7
TransIT®-LT1 Transfection Reagent	Mirus Bio LLC, Madison, WI, USA	MR-MIR2300
Human RTK Phosphorylation Array C1	RayBiotech Life, Inc., Norcross, GA, USA	AAH-PRTK-1-2
FITC Annexin V Apoptosis Detection Kit	BD Bioscience, Becton Drive Franklin Lakes, NJ, USA	CAT 556547
3,3'-Dihexyloxacarbocyanine iodide, DIOC_6_	Sigma-Aldrich	CAS 53213-82-4
Propidium Iodide (PI)	Sigma-Aldrich	25535-16-4
Human RTK Phosphorylation Array C1	RayBiotech Life, Inc., Norcross, GA, USA	AAH-PRTK-1-2
CaspGLOW™ Fluorescein Active Caspase-3	BioVision, San Francisco, CA, USA	K183
CaspGLOW™ Fluorescein Active Caspase-8	BioVision	K188
CaspGLOW™ Fluorescein Active Caspase-9	BioVision	K189

**Table 2:** Primary antibodies used in this study.

Antibodies	Company	Product no.
LYN (Tyr 507)	Cell Signaling Technology, Danvers, MA,USA	#2731
LYN	Cell Signaling Technology	#2732
ERK1/2 (Thr202/Try204)	Elabscience, Houston, TX, USA	E-AB-21303
ERK1/2	Elabscience	E-AB-31373
STAT3 (Tyr705)	Cell Signaling Technology	#9145
STAT3	Cell Signaling Technology	#9139
NF-κB (Ser 536)	Cell Signaling Technology	#3033
NF-κB	Cell Signaling Technology	#8242
VEGF-A	Abcam, Cambridge, UK	ab46154
MMP-2	Invitrogen, Waltam, MA,USA	MA5-13590
MMP-9	Invitrogen	PA5-13199
E-cadherin	Cell Signaling Technology	#3195
N-cadherin	Cell Signaling Technology	#13116
Snail1	Cell Signaling Technology	#3879
Slug	Cell Signaling Technology	#9585
ZEB1	Cell Signaling Technology	#70512
ZEB2	Cell Signaling Technology	#97885
TWIST	Cell Signaling Technology	#46702
β-actin	Elabscience	E-AB-20058
Fas-FITC	BD Bioscience	556640
Fas-PE	BD Bioscience	564261

**Table 3:** 10 minutes' bio-distribution of ^68^Ga-CC12 in normal mice and GBM8401 bearing mice are presented as follow.

10 min %ID/g	Normal mice	Tumor mice
Blood	1.19	1.46
R Brain (Tumor)	0.40	35.80
L Brain	0.21	0.33
Heart	1.60	1.30
Kidney	13.28	22.83
Liver	38.83	53.12
Spleen	109.98	142.10
Colon	2.30	2.46
Bladder	1.75	2.35
Skin	3.14	4.00
Stomach	1.32	2.14
Bone	10.35	8.95
Lung	22.03	34.80

**Table 4:** 1 hr's bio-distribution of ^68^Ga-free and ^68^Ga-CC12 in normal mice, GBM8401 and RT whole brain exposure mice bearing mice are presented as follow. (AVG=Average; SEM=standard error)

1 hr (average±SEM)	^68^Ga-free %ID/g		^68^Ga-CC12 %ID/g		
Organ/Model	Normal		Normal	GBM8401 tumor	RT brain (6 Gy)
Blood	4.32±2.32		3.52±2.5	4.95±0.24	3.15±2.41
Whole Brain	0.26±0.07		33.56±0.99	36.84±2.52	33.95±0.32
Heart	1.02±0.01		1.48±0.1	1.14±0.15	0.8±0.03
Kidney	59.33±2.39		51.35±0.99	49.33±0.81	62.07±4.92
Liver	6.99±0.03		7.1±0.1	6.21±1.49	7.1±0.15
Spleen	2.1±1.38		2.92±0.21	1.92±0.36	2.14±0.23
Bladder	150.44±3.68		146.11±1.82	140.29±13.11	155.98±6.23
Skin	1.96±0.08		2.56±0.45	1.53±0.01	1.98±0.14
Stomach	1.24±0.09		1.52±0.21	1.22±0.02	1.52±0.02
Bone	1.87±0.15		3.3±0.84	2.17±0.26	2.27±1.32
Lung	5.37±0.33		5.37±0.75	5.11±0	5.44±0.75
Intestines	1.18±0.84		1.13±0.37	1.02±0.13	1.09±0.12
Muscle	0.79±0.04		0.75±0.09	0.62±0.08	0.74±0.06

**Table 5:** 1 hr's and 3 hr's bio-distribution of ^68^Ga-CC12 in normal mice, GBM8401 and RT whole brain exposure mice bearing mice are presented as follow. (AVG=Average; SEM=standard error)

(average±SEM)	^68^Ga-CC12 1 hr %ID/g	^68^Ga-CC12 3 hr %ID/g
Organ/Model	Normal	Normal
Blood	3.52±2.5	5.24±1.2
Whole Brain	33.56±0.99	1.23±0.24
Heart	1.48±0.1	1.66±0.04
Kidney	51.35±0.99	43.39±1.62
Liver	7.1±0.1	5.68±0.29
Spleen	2.92±0.21	0.21±0.01
Bladder	146.11±1.82	69.47±0.65
Skin	2.56±0.45	0.76±0.03
Stomach	1.52±0.21	0.77±0.15
Bone	3.3±0.84	0.99±0.31
Lung	5.37±0.75	0.55±0.01
Intestines	1.13±0.37	0.39±0.06
Muscle	0.75±0.09	0.06±0

## References

[1] Sung H, Ferlay J, Siegel RL, Laversanne M, Soerjomataram I, Jemal A (2021). Global Cancer Statistics 2020: GLOBOCAN Estimates of Incidence and Mortality Worldwide for 36 Cancers in 185 Countries. CA Cancer J. Clin.

[2] Sze G (1993). Diseases of the intracranial meninges: MR imaging features. American Journal of Roentgenology.

[3] Stupp R, Mason WP, Van Den Bent MJ, Weller M, Fisher B, Taphoorn MJ (2005). Radiotherapy plus concomitant and adjuvant temozolomide for glioblastoma. N Engl J Med.

[4] Wen PY, Kesari S (2008). Malignant gliomas in adults. N Engl J Med.

[5] Amarandi R-M, Ibanescu A, Carasevici E, Marin L, Dragoi B (2022). Liposomal-Based Formulations: A Path from Basic Research to Temozolomide Delivery Inside Glioblastoma Tissue. Pharmaceutics.

[6] Wilson TA, Karajannis MA, Harter DH (2014). Glioblastoma multiforme: State of the art and future therapeutics. Surg Neurol Int.

[7] Verhaak RG, Hoadley KA, Purdom E, Wang V, Qi Y, Wilkerson MD (2010). Integrated genomic analysis identifies clinically relevant subtypes of glioblastoma characterized by abnormalities in PDGFRA, IDH1, EGFR, and NF1. Cancer cell.

[8] René CA, Parks RJ (2021). Delivery of Therapeutic Agents to the Central Nervous System and the Promise of Extracellular Vesicles. Pharmaceutics.

[9] Manciu FS, Guerrero J, Bennet KE, Chang S-Y, Rahman M, Martinez Lopez LV (2022). Assessing Nordihydroguaiaretic Acid Therapeutic Effect for Glioblastoma Multiforme. Sensors.

[10] Mao L, Deng WW, Yu GT, Bu LL, Liu JF, Ma SR (2017). Inhibition of SRC family kinases reduces myeloid-derived suppressor cells in head and neck cancer. Int J Cancer.

[11] Su R, Zhang J (2020). Oncogenic role of LYN in human gastric cancer via the Wnt/β-catenin and AKT/mTOR pathways. Exp Ther Med.

[12] Kim YJ, Hong S, Sung M, Park MJ, Jung K, Noh KW (2016). LYN expression predicts the response to dasatinib in a subpopulation of lung adenocarcinoma patients. Oncotarget.

[13] Stettner MR, Wang W, Nabors LB, Bharara S, Flynn DC, Grammer JR (2005). Lyn kinase activity is the predominant cellular SRC kinase activity in glioblastoma tumor cells. Cancer Res.

[14] Patel A, Sabbineni H, Clarke A, Somanath PR (2016). Novel roles of Src in cancer cell epithelial-to-mesenchymal transition, vascular permeability, microinvasion and metastasis. Life Sci.

[15] Zhong L, Li Y, Xiong L, Wang W, Wu M, Yuan T (2021). Small molecules in targeted cancer therapy: advances, challenges, and future perspectives. Signal Transduction and Targeted Therapy.

[16] Lawal B, Wang Y-C, Wu ATH, Huang H-S (2021). Pro-Oncogenic c-Met/EGFR, Biomarker Signatures of the Tumor Microenvironment are Clinical and Therapy Response Prognosticators in Colorectal Cancer, and Therapeutic Targets of 3-Phenyl-2H-benzo[e][1,3]-Oxazine-2,4(3H)-Dione Derivatives. Front Pharmacol.

[17] Lee JC, Wu ATH, Chen JH, Huang WY, Lawal B, Mokgautsi N (2020). HNC0014, a Multi-Targeted Small-Molecule, Inhibits Head and Neck Squamous Cell Carcinoma by Suppressing c-Met/STAT3/CD44/PD-L1 Oncoimmune Signature and Eliciting Antitumor Immune Responses. Cancers.

[18] Mokgautsi N, Wang Y-C, Lawal B, Khedkar H, Sumitra MR, Wu AT (2021). Network Pharmacological Analysis through a Bioinformatics Approach of Novel NSC765600 and NSC765691 Compounds as Potential Inhibitors of CCND1/CDK4/PLK1/CD44 in Cancer Types. Cancers.

[19] Lee CC, Huang KF, Lin PY, Huang FC, Chen CL, Chen TC (2012). Synthesis, antiproliferative activities and telomerase inhibition evaluation of novel asymmetrical 1,2-disubstituted amidoanthraquinone derivatives. Eur J Med Chem.

[20] Huang HS, Huang KF, Li CL, Huang YY, Chiang YH, Huang FC (2008). Synthesis, human telomerase inhibition and anti-proliferative studies of a series of 2,7-bis-substituted amido-anthraquinone derivatives. Bioorg Med Chem.

[21] Siddamurthi S, Gutti G, Jana S, Kumar A, Singh SK (2020). Anthraquinone: a promising scaffold for the discovery and development of therapeutic agents in cancer therapy. Future Med Chem.

[22] Tornabene E, Brodin B (2016). Stroke and Drug Delivery-In Vitro Models of the Ischemic Blood-Brain Barrier. J Pharm Sci.

[23] Guanglei L, Melissa JS, Limary MC, Shi ZD, Ji XY, John MT (2010). Permeability of endothelial and astrocyte cocultures: in vitro blood-brain barrier models for drug delivery studies. Ann Biomed Eng.

[24] Chiang IT, Liu YC, Liu HS, Ali AAA, Chou SY, Hsu TI (2022). Regorafenib Reverses Temozolomide-Induced CXCL12/CXCR4 Signaling and Triggers Apoptosis Mechanism in Glioblastoma. Neurotherapeutics.

[25] Hsu FT, Liu HS, Ali AAA, Tsai PH, Kao YC, Lu CF (2018). Assessing the selective therapeutic efficacy of superparamagnetic erlotinib nanoparticles in lung cancer by using quantitative magnetic resonance imaging and a nuclear factor kappa-B reporter gene system. Nanomedicine.

[26] Trott O, Olson AJ (2010). AutoDock Vina: improving the speed and accuracy of docking with a new scoring function, efficient optimization, and multithreading. J Comput Chem.

[27] Marcus D Hanwell DEC, David C Lonie, Tim Vandermeersch, Eva Zurek, Geoffrey R Hutchison (2012). Avogadro: An advanced semantic chemical editor, visualization, and analysis platform. J Cheminformatics.

[28] Lawal B, Liu Y-L, Mokgautsi N, Khedkar H, Sumitra MR, Wu AT (2021). Pharmacoinformatics and preclinical studies of nsc765690 and nsc765599, potential stat3/cdk2/4/6 inhibitors with antitumor activities against nci60 human tumor cell lines. Biomedicines.

[29] Lawal B, Lee CY, Mokgautsi N, Sumitra MR, Khedkar H, Wu AT (2021). mTOR/EGFR/iNOS/MAP2K1/FGFR/TGFB1 Are Druggable Candidates for N-(2, 4-Difluorophenyl)-2', 4'-Difluoro-4-Hydroxybiphenyl-3-Carboxamide (NSC765598), With Consequent Anticancer Implications. Front Oncol.

[30] Lawal B, Lee C-Y, Mokgautsi N, Sumitra MR, Khedkar H, Wu ATH (2021). mTOR/EGFR/iNOS/MAP2K1/FGFR/TGFB1 Are Druggable Candidates for N-(2,4-Difluorophenyl)-2′,4′-Difluoro-4-Hydroxybiphenyl-3-Carboxamide (NSC765598), With Consequent Anticancer Implications. Front Oncol.

[31] Visualizer DS BIOVIA, Dassault Systèmes, BIOVIA Workbook, Release 2020; BIOVIA Pipeline Pilot, Release 2020, San Diego: Dassault Systèmes. 2020.

[32] Liu H, Wang L, Lv M, Pei R, Li P, Pei Z (2014). AlzPlatform: an Alzheimer's disease domain-specific chemogenomics knowledgebase for polypharmacology and target identification research. J Chem Inf Model.

[33] Salvesen GS, Dixit VM (1997). Caspases: intracellular signaling by proteolysis. Cell.

[34] Pearson GW (2019). Control of Invasion by Epithelial-to-Mesenchymal Transition Programs during Metastasis. J Clin Med.

[35] Rivera AL, Pelloski CE, Gilbert MR, Colman H, De La Cruz C, Sulman EP (2010). MGMT promoter methylation is predictive of response to radiotherapy and prognostic in the absence of adjuvant alkylating chemotherapy for glioblastoma. Neuro Oncol.

[36] Kitange GJ, Carlson BL, Schroeder MA, Grogan PT, Lamont JD, Decker PA (2009). Induction of MGMT expression is associated with temozolomide resistance in glioblastoma xenografts. Neuro Oncol.

[37] Valdés-Rives SA, Casique-Aguirre D, Germán-Castelán L, Velasco-Velázquez MA, González-Arenas A (2017). Apoptotic Signaling Pathways in Glioblastoma and Therapeutic Implications. Biomed Res Int.

[38] Kataoka T (2005). The caspase-8 modulator c-FLIP. Crit Rev Immunol.

[39] Day B, Stringer B, Charmsaz S, Jamieson P, Ensbey K, Carter J (2011). ELK4 neutralization sensitizes glioblastoma to apoptosis through downregulation of the anti-apoptotic protein Mcl-1. Neuro Oncol.

[40] Wei J, Wang Z, Wang W, Liu X, Wan J, Yuan Y (2021). Oxidative Stress Activated by Sorafenib Alters the Temozolomide Sensitivity of Human Glioma Cells Through Autophagy and JAK2/STAT3-AIF Axis. Front Cell Dev Biol.

[41] Wong AJ, Ruppert JM, Bigner SH, Grzeschik CH, Humphrey PA, Bigner DS (1992). Structural alterations of the epidermal growth factor receptor gene in human gliomas. Proc Natl Acad Sci USA.

[42] Wheeler SE, Morariu EM, Bednash JS, Otte CG, Seethala RR, Chiosea SI (2012). Lyn kinase mediates cell motility and tumor growth in EGFRvIII-expressing head and neck cancer. Clin Cancer Res.

[43] Li Z, Liao J, Yang Z, Choi EY, Lapidus RG, Liu X (2019). Co-targeting EGFR and IKKβ/NF-κB signalling pathways in head and neck squamous cell carcinoma: a potential novel therapy for head and neck squamous cell cancer. Br J Cancer.

[44] Bommhardt U, Schraven B, Simeoni L (2019). Beyond TCR Signaling: Emerging Functions of Lck in Cancer and Immunotherapy. Int J Mol Sci.

[45] Feng H, Hu B, Jarzynka Michael J, Li Y, Keezer S, Johns Terrance G (2012). Phosphorylation of dedicator of cytokinesis 1 (Dock180) at tyrosine residue Y722 by Src family kinases mediates EGFRvIII-driven glioblastoma tumorigenesis. Proc Natl Acad Sci USA.

[46] Liang X, He X, Li Y, Wang J, Wu D, Yuan X (2019). Lyn regulates epithelial-mesenchymal transition in CS-exposed model through Smad2/3 signaling. Respiratory Research.

[47] Guarino M (2010). Src signaling in cancer invasion. J Cell Physiol.

[48] Wu HT, Zhong H-T, Li GW, Shen JX, Ye QQ, Zhang M-L (2020). Oncogenic functions of the EMT-related transcription factor ZEB1 in breast cancer. J Transl Med.

[49] Serrano-Gomez SJ, Maziveyi M, Alahari SK (2016). Regulation of epithelial-mesenchymal transition through epigenetic and post-translational modifications. Mol Cancer.

[50] Gonzalez DM, Medici D (2014). Signaling mechanisms of the epithelial-mesenchymal transition. Sci Signal.

[51] Yeh Y-C, Lawal B, Hsiao M, Huang T-H, Huang C-YF (2021). Identification of NSP3 (SH2D3C) as a Prognostic Biomarker of Tumor Progression and Immune Evasion for Lung Cancer and Evaluation of Organosulfur Compounds from Allium sativum L. as Therapeutic Candidates. Biomedicines.

[52] Meng X-Y, Zhang H-X, Mezei M, Cui M (2011). Molecular docking: a powerful approach for structure-based drug discovery. Curr Comput Aided Drug Des.

[53] Chen J-H, Wu ATH, Lawal B, Tzeng DTW, Lee J-C, Ho C-L (2021). Identification of Cancer Hub Gene Signatures Associated with Immune-Suppressive Tumor Microenvironment and Ovatodiolide as a Potential Cancer Immunotherapeutic Agent. Cancers.

[54] Yuan F, Chen Y, Dellian M, Safabakhsh N, Ferrara N, Jain RK (1996). Time-dependent vascular regression and permeability changes in established human tumor xenografts induced by an anti-vascular endothelial growth factor/vascular permeability factor antibody. Proc Natl Acad Sci USA.

