# Supplementary material for: A Novel Isotope-labeled Small Molecule Probe CC12 for Anti-glioma via Suppressing LYN-mediated Progression and Activating Apoptosis Pathways
Source: Int J Biol Sci. 2023 Jun 19;19(10):3209–25. doi: 10.7150/ijbs.82266 (PMC10321274; doi:10.7150/ijbs.82266)
Supplement: Supplementary file 1 — Supplementary methods and figures. [file ijbsv19p3209s1.pdf]

A Novel Isotope-labeled Small Molecule Probe CC12 for Anti-glioma via Suppressing LYN-mediated Progression and Activating Apoptosis Pathways

Supplementary methods and figures

1.1 Flow cytometric analysis

GBM8401 and T98G cells were seeded in six-well plates (10^5 cells/well) overnight and treated with CC12 for 48 h. After treatment, cells were harvested, fixed, counted, and stained with different antibodies or dyes as previously described [1, 2]. Staining reagents are listed as follows: FITC-DEVD-FMK (cleaved caspase-3), FITC-IETD-FMK (cleaved caspase-8), FITC-VAD-FMK (cleaved caspase-9), Fas-PE, Fas-FITC, $DIOC_6$ (for the mitochondrial membrane potential), propidium iodide (PI), and FITC Annexin V Apoptosis Detection Kit (BD Bioscience, Franklin Lakes, NJ, USA). Fluorescence signals emitted from stained cells were determined by flow cytometry (NovoCyte, Agilent Technologies, Santa Clara, CA, USA) and quantified by FlowJo software (vers. 7.6.1; FlowJo LLC, Ashland, OR, USA).

1.2 Invasion and migration (metastasis) analysis

GBM8401 and T98G cells were seeded in 10-cm plates (10^6 cells/plate) overnight and treated with CC12 for 48 h. After treatment, 10^5 cells were harvested, counted, and seeded into transwells with 30% Matrigel (invasion) or without (migration). Cells were allowed to migrate or invade for 48 h, fixed with fixation buffer (methanol: acetic acid = 3: 1) and stained with 0.1% crystal violet for 15 min [3]. GBM8401 cells after small hairpin (sh)RNA-knockdown and overexpression were also used to performed the invasion experiment. Transwell membranes were then isolated for visualization with a Nikon ECLIPSE Ti-U microscope (Tokyo, Japan) and quantified by ImageJ software vers. 1.50 (National Institutes of Health (NIH), Bethesda, MD, USA).

1.3 Western blot analysis

GBM8401 and T98G cells were seeded in 10-cm plates (3×10^6 cells/plate) overnight and treated with CC12 for 48 h. After treatment, cells were harvested, lysed with NP-40 buffer (containing a phosphatase and proteinase inhibitor cocktail), and quantified by the Bradford assay. Proteins were then separated by sodium

dodecylsulfate (SDS)-polyacrylamide gel electrophoresis (PAGE) (8%~15%), transferred onto polyvinylidene difluoride (PVDF) membranes, stained with the primary antibody, followed with the secondary antibody, visualized by a chemiluminescent horseradish peroxidase (HRP) substrate, and finally detected by UVP ChemiDoc-ItTM (Analytik Jena, Jena, Germany) as previously described [4]. Primary and secondary antibodies are listed in Table 2.

1.4 Generation of lentiviral particles for knockdown and overexpression of LYN

Three lentiviral transfer vectors, viz., pGFP-C-shLenti (TR30023) targeting different regions of LYN (Gene ID = 4067; TL320407A and TL320407B) and a non-effective 29-mer scrambled shRNA (pGFP-C-shLenti Vector; TR30021) were ordered from OriGene. Lentiviral particles were produced by transfecting HEK 293T cells with a trans-packaging plasmid mix (shRNA vector, packaging plasmid: pCMV-ΔR8.91 and envelope plasmid: pMD.G, a VSV-G expressing plasmid) and TransIT®-LT1 Transfection Reagent (Mirus Bio LLC, Madison, WI, USA). HEK 293T cells were incubated for 18 h. The medium was changed and replaced with 10 mL bovine serum albumin (BSA)-containing medium (1% BSA) for 18 h. Media were harvested after incubation for 40 and 64 h. Lentivirus packages were stored at -80 °C for long-term storage. The human LYN open reading frame (NM_001111097) was inserted into the pLenti-C-mGFP-P2A-Puro (PS100093) vector ordered from OriGene. Lentiviral particles were produced in HEK 293T cells by transfecting cells with the pLenti-C-mGFP LYN vector (SR175449) and trans-packaging plasmid mix (packaging plasmid: pCMV-ΔR8.91 and envelope plasmid: pMD.G, aVSV-G-expressing plasmid) and using the TransIT®-LT1 Transfection Reagent.

1.5 Knockdown and overexpression of LYN

GBM8401 cells were virally transfected with pGFP-C-shLenti (scrambled)/pGFP-C-shLenti-LYN and pLenti-C-mGFP-P2A-Puro (MOCK)/pLenti-C-mGFP-P2A-Puro-LYN (overexpression). GBM8401 cells at 50% confluence were incubated for 24 h in a 1:9 dilution of the virus package with 4 μg/ml polybrene. After 24 h of recovery in normal culture medium with no virus, cells were selected with 1 μg/ml puromycin for 7 days before being used in subsequent experiments.

1.6 NF-κB transfection and *in vivo* reporter gene assay

GBM8401 cells were transfected with pNF-κB-luc2, selected as a stable clone by 200 μg/ml hygromycin B and named GBM8401/*NF-κB-luc2* [5]. For animal experiments, the GBM8401/*NF-κB-luc2* signal from mice brains was detected by the IVIS200 Imaging System after intraperitoneally injecting 150 mg/kg body weight D-luciferin. The signal intensity of NF-κB in mice brains was quantified through drawing regions of interest (ROIs) with Living Image software.

1.7 Protein kinase array

The human RTK Phosphorylation Array C1 was purchased from RayBio® (RayBiotech Life, Norcross, GA, USA). GBM8401 cells were treated with 0 and 20 μM of CC12 for 48 h, and proteins were extracted to perform the experiment. Array membranes were incubated with 1 ml (250 μg) of protein lysis of GBM8401 cells overnight at 4 °C. After washing the membranes, they were incubated with biotin-conjugated antibodies (mouse Biotinylated Antibody Cocktail) overnight. Then, membranes were incubated with streptavidin-HRP for 2 h and a chemiluminescence reagent mix for 2 min. Immunoreactive dots on the membranes were detected by a chemiluminescent image system (ChemiDoc-It 515, UVP, Upland, CA, USA).

1.8 GeneMANIA and GEPIA analysis

The keywords of LYN, STAT3, MAPK1/2, and NF-κB were filled in the geneMANIA system for identifying the correlation of each other in Homo sapiens. We utilized RNA data sets from The Cancer Genome Atlas open-source collection, which can be accessed through the GEPIA website. GEPIA (Gene Expression Profiling Interactive Analysis) is an online resource for gene expression analysis and survival relation with specific gene. Here, we used input LYN, HGFR, TKY2 gene that we illustrated from CC12 protein kinase array.

1.9 Preparation of ^{68}Ga radioisotope labeled CC-12.

To prepare ^{68}Ga labeled CC-12, first 1 mL of ^{68}Ga is added to CC-12. The mixture is then agitated on a 300 rpm shaker at 37 °C for 10-30 minutes to produce ^{68}Ga-CC-12. To purify the mixture, a centrifuge is used at 14,000 rpm for 10 minutes with a 10k centrifuge tube (Sartorius Vivaspin®, Göttingen, Germany). The

supernatant liquid is removed, and the particles are suspended in normal saline and repeated once. Finally, the radiolabeling yield and radiochemical purity are checked using an instant thin layer chromatography system (AR-2000 radio-TLC Imaging Scanner, Bioscan Inc., Poway, CA, USA).

1.10 *In vivo* Nano-SPECT/CT scan

For the investigation of *in vivo* biodistribution of ^{68}Ga-CC-12, the signal of it (Activity equivalent to 37 MBq) in mice were obtained by using Positron Emission Tomography - Computed Tomography (PET/CT). The ^{68}Ga-CC-12 particles in 100 μL of normal saline was injected through intravenous injection. The signal of ^{68}Ga-CC-12 in the mouse brain was assessed immediately using a NanoScan PET/CT scanner system (Mediso, Arlington, VA, USA) following injection. Following the capture of the PET images, CT images were acquired (X-ray source: 70 kV, 1 mA; 256 projections) at the same position. The images were fusion by using InVivoScope® software. To ensure proper imaging, the mice were anesthetized with inhalation of isoflurane (3-4%) during scanning.

1.11 Biodistribution of ^{68}Ga-CC-12

The biodistribution study of ^{68}Ga-free or ^{68}Ga-CC-12 in mice was conducted on normal mice, mice with GBM8401 tumor (150~200 mm^3), 6Gy RT whole brain exposure model (n=3), respectively. Mice were anesthetized with isoflurane and injected with ^{68}Ga-CC-12 (3.7 MBq) through the intra-carotid artery. After 10 min, 1 hr or 3 hr min injection, mice were sacrificed by CO_2. The organs were excised, weighed, and assayed for radioactivity by gamma counter (1470Wizard, PerkinElmer, Waltham, MA, USA). The tissue activity was displayed as percentage injected dose per gram of tissue (%ID/g).

1.12 Hematoxylin and eosin (H&E) staining and immunohistochemical (IHC) staining

Mice tumors were isolated for staining on day 21. The staining procedure was described in previous work [6]. Images were scanned with a TissueFAXS Tissue-Gnostics Axio Observer Z1 microscope (TissueGnostics) and quantified by the ImageJ IHC tool box developed by the NIH [7].

1.13 BBB permeation algorithm

The BBB permeability graph was built using the ALzPlatform, a BBB permeation algorithm that applies support vector machine (SVM) and LiCABEDS algorithms to simulate drug density that pass the BBB. The algorithm used the physicochemical properties and structural coordinate of the drugs to simulate the peak density corresponding to the scores of the drugs BBB permeability.

2. Supplementary data

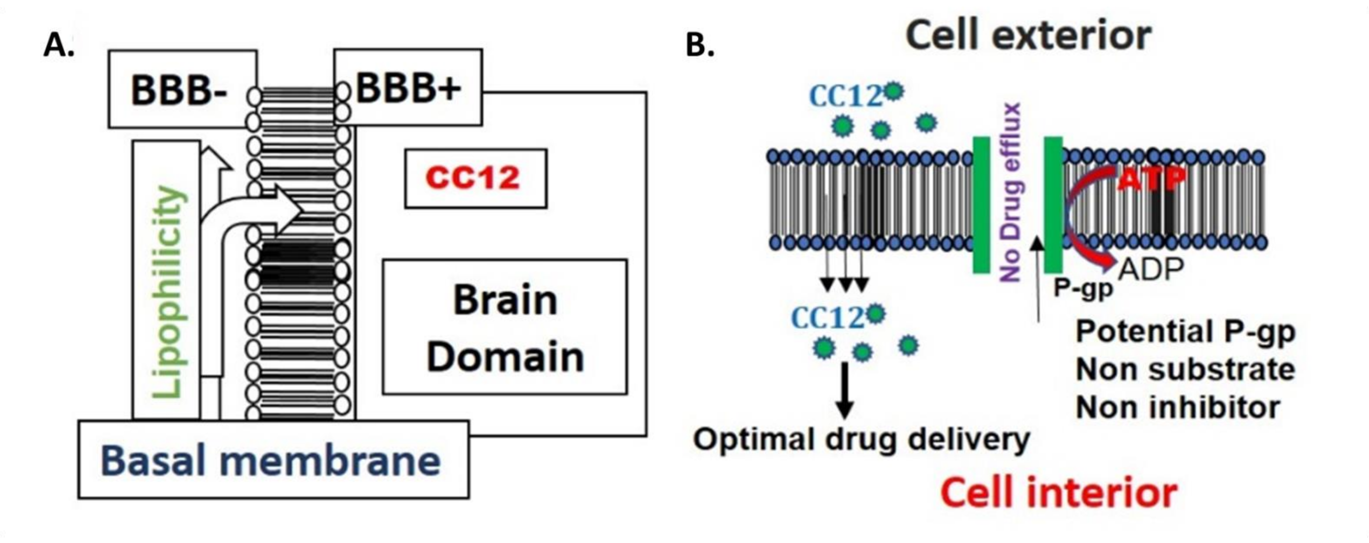

Supplementary figure 1. Figure demonstrated non-substrability and non-inhibitor tendencies towards P-gp of CC12.

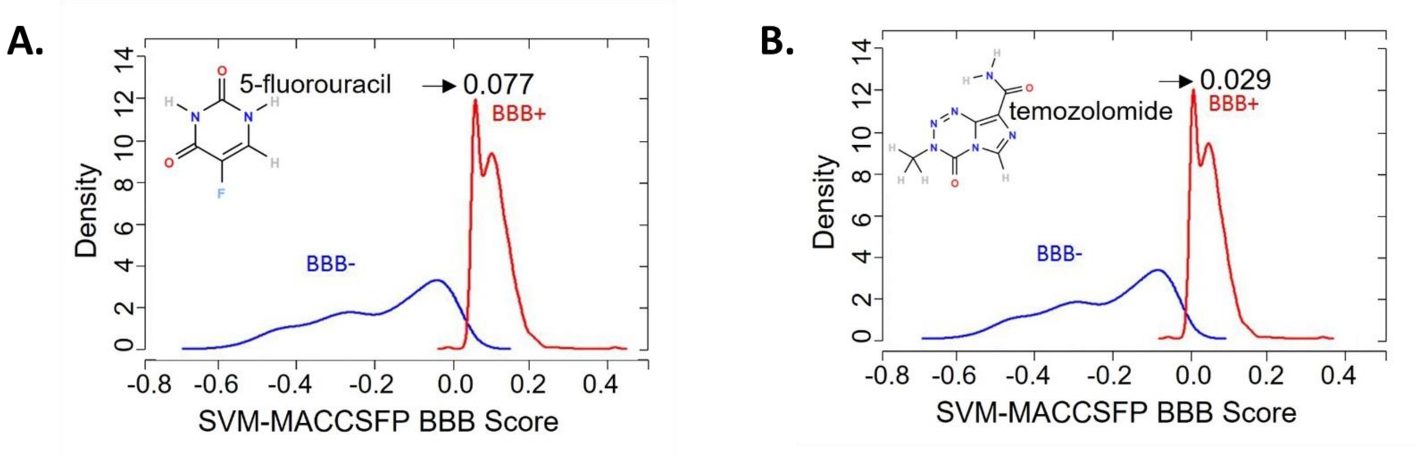

Supplementary figure 2. In silico BBB permeability curve of (A) 5-FU and (B) TMZ based on support vector machine (SVM) and LiCABEDS algorithms is displayed.

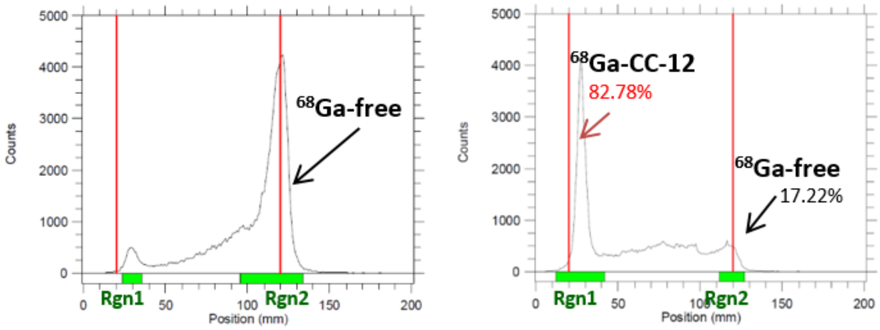

Reg	(mm) Start	(mm) Stop	(mm) Centroid	RF	Region Counts	Region CPM	% of Total	% of ROI
Rgn 1	23.9	35.7	29.6	0.096	4492.0	4492.0	13.63	5.63
Rgn 2	95.5	133.5	115.6	0.956	75362.0	75362.0	228.67	94.37
2 Peaks					79854.0	79854.0	242.30	100.00

Reg	(mm) Start	(mm) Stop	(mm) Centroid	RF	Region Counts	Region CPM	% of Total	% of ROI
Rgn 1	12.2	42.0	28.3	0.083	32580.0	32580.0	53.55	82.78
Rgn 2	111.8	127.2	117.9	0.979	6779.0	6779.0	11.14	17.22
2 Peaks					39359.0	39359.0	64.69	100.00

Supplementary figure 3. The ^{68}Ga labeling efficacy on CC12 is displayed.

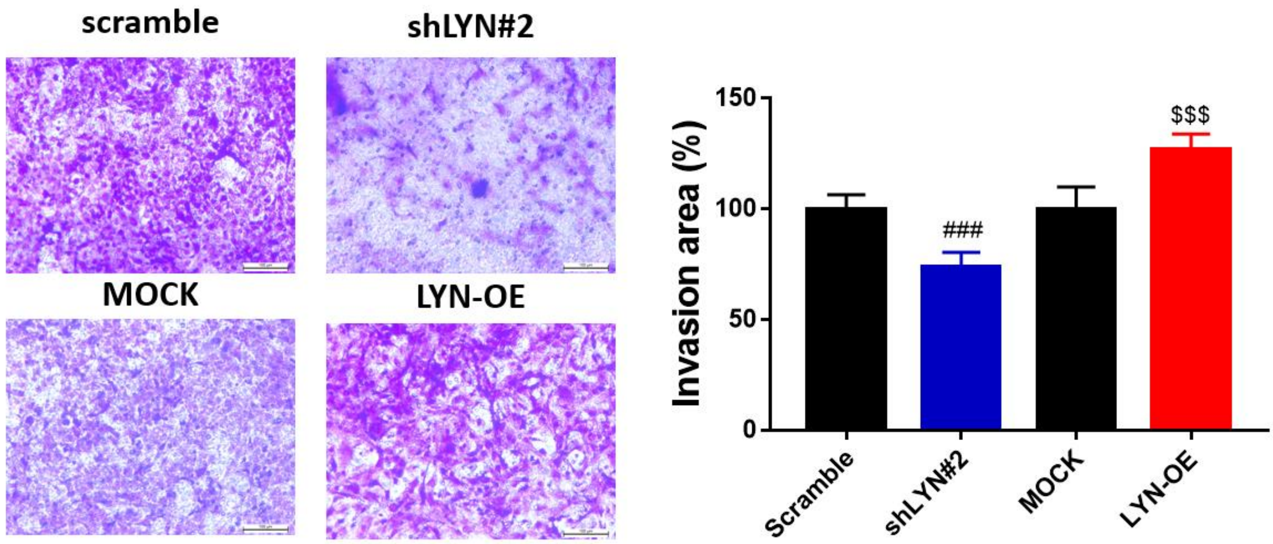

Supplementary figure 4. Invasion results in LYN knockdown and overexpression GBM8401 cells.

Supported data

3.1 Full blot images of Figure 2A

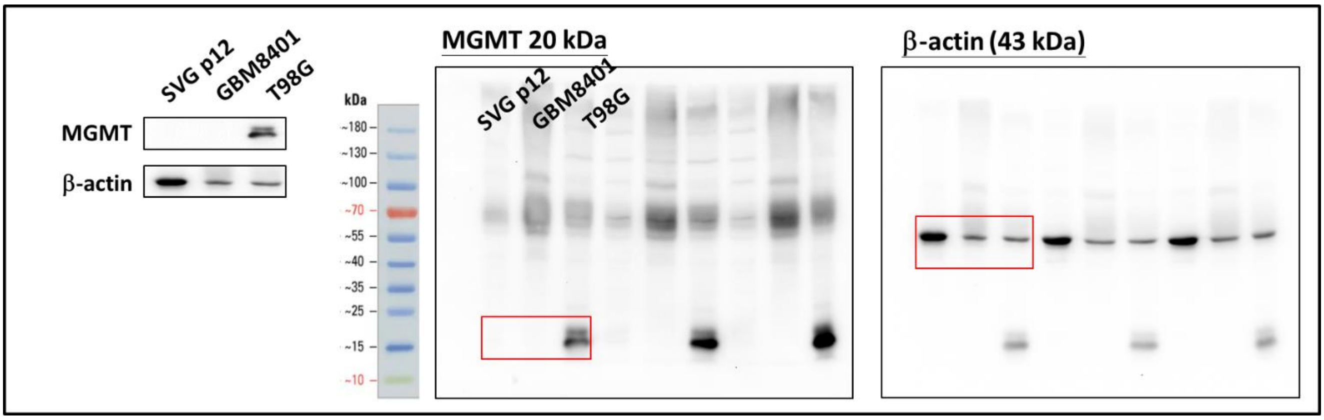

3.2 Full blot images of Figure 3D

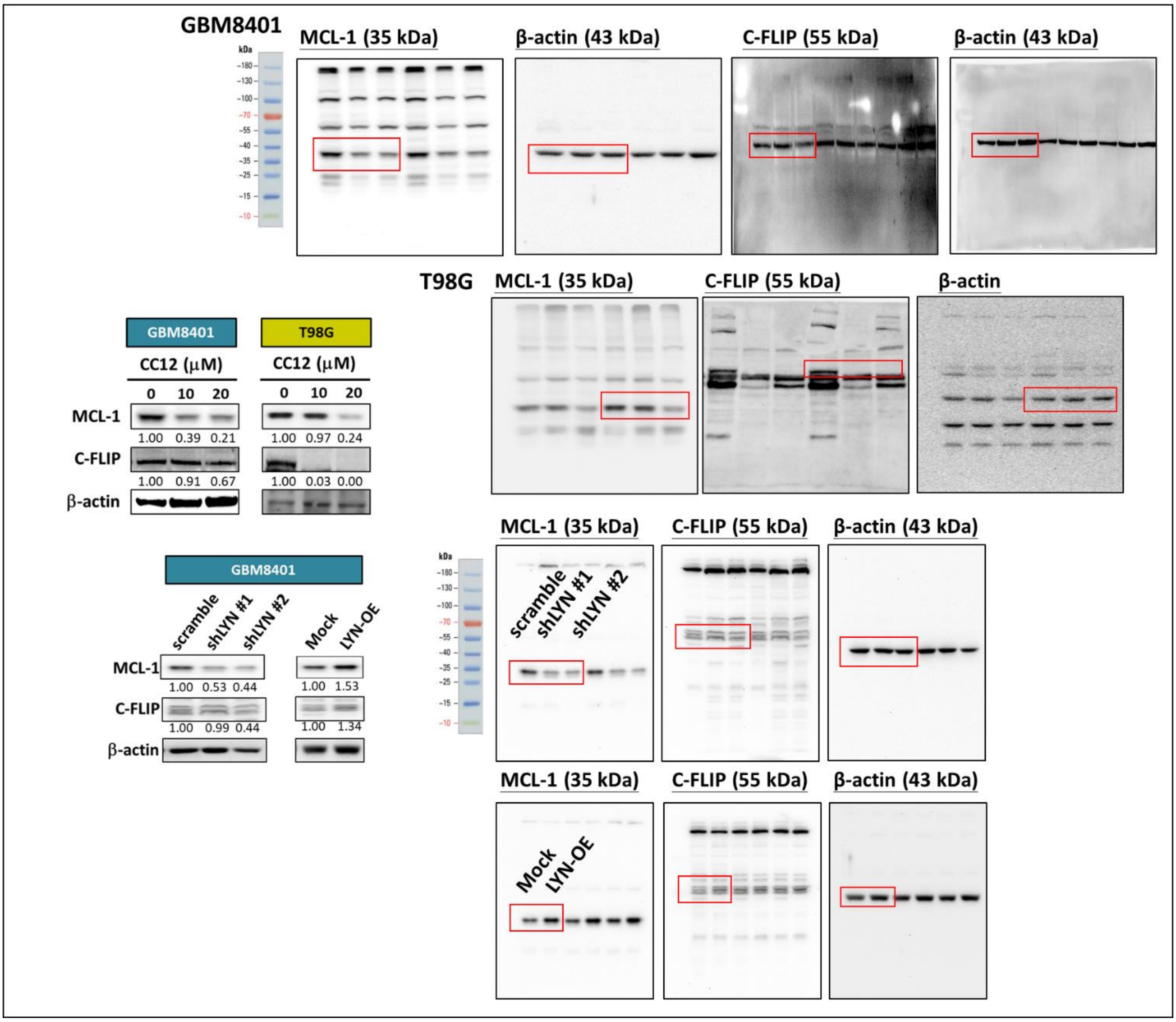

3.3 Full blot images of Figure 4F

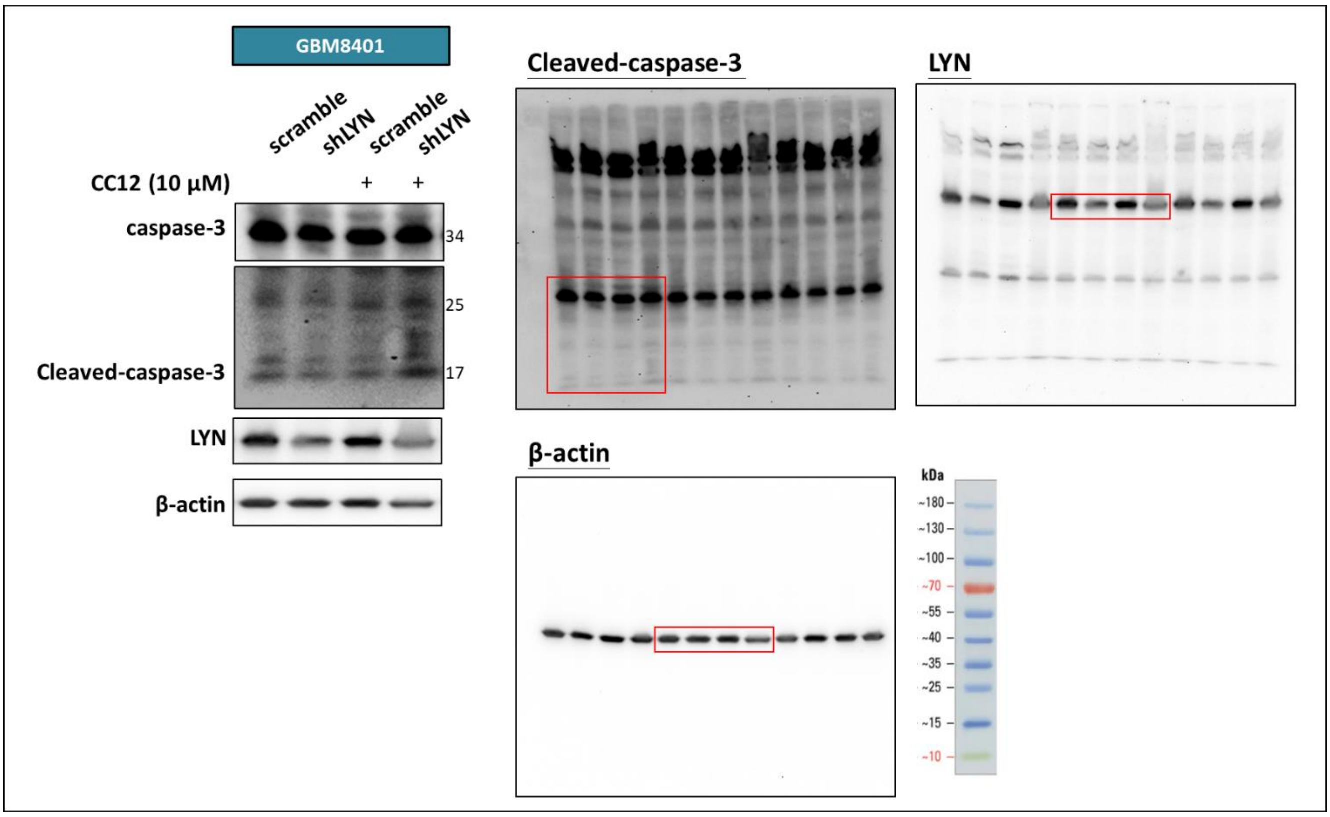

GBM8401
scramble
shLYN
scramble
shLYN
CC12 (10 μM)
+
+
caspase-3
34
25
Cleaved-caspase-3
17
LYN
β-actin
Cleaved-caspase-3
LYN
β-actin
kDa
~180
~130
~100
~70
~55
~40
~35
~25
~15
~10

3.4 Full blot images of Figure 4H left panel

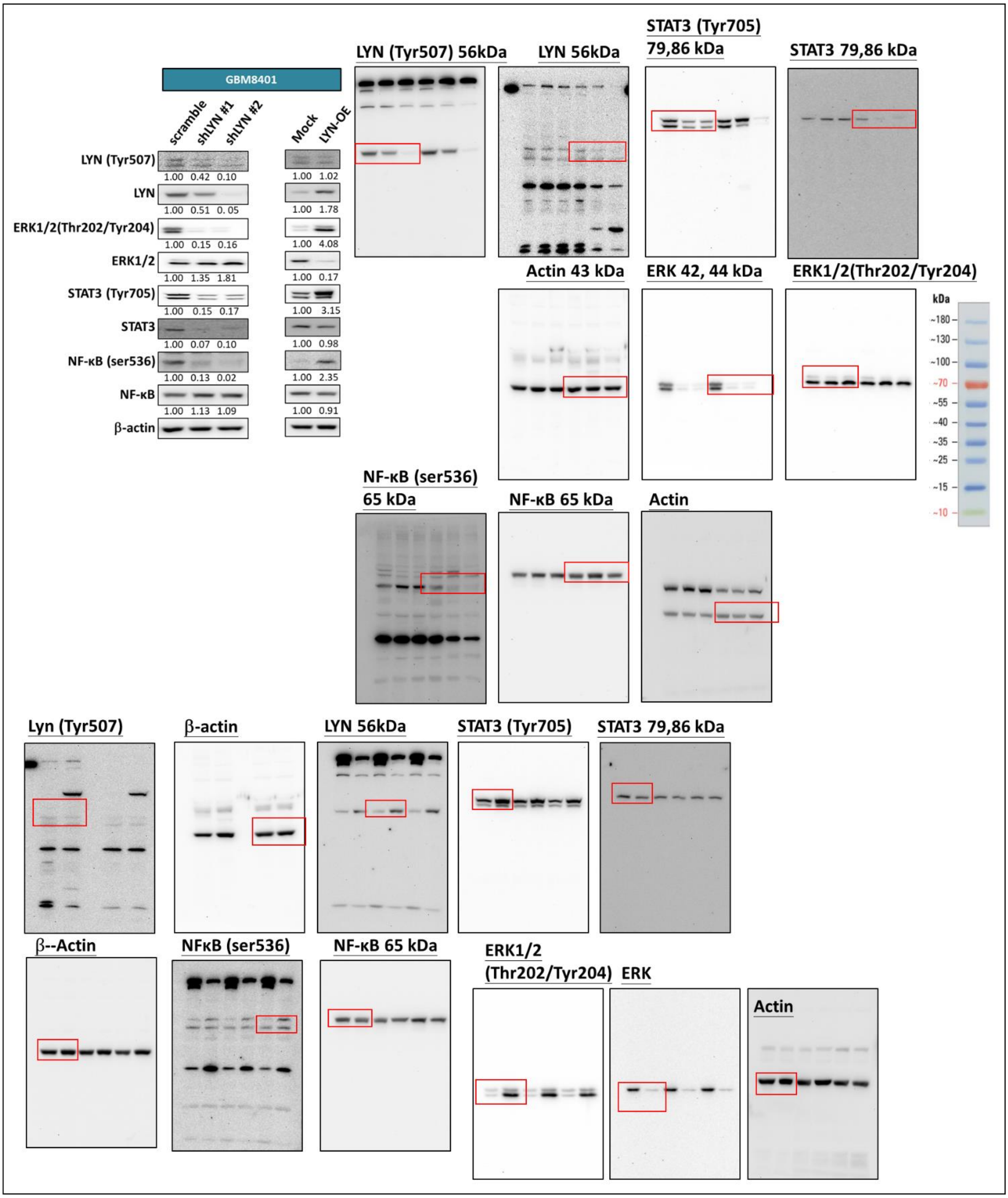

3.4 Full blot images of Figure 4I panel

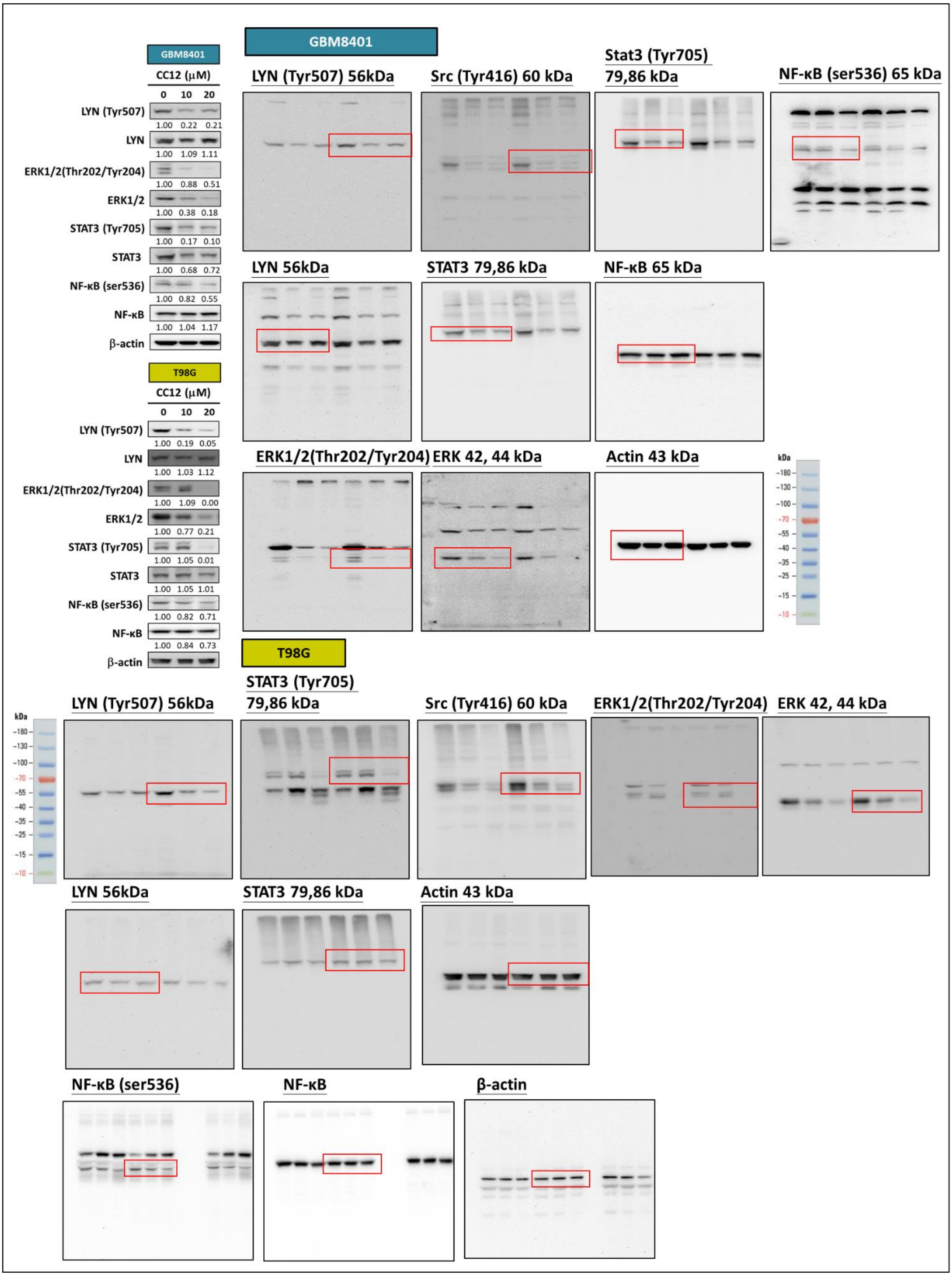

3.5 Full blot images of Figure 5D right panel

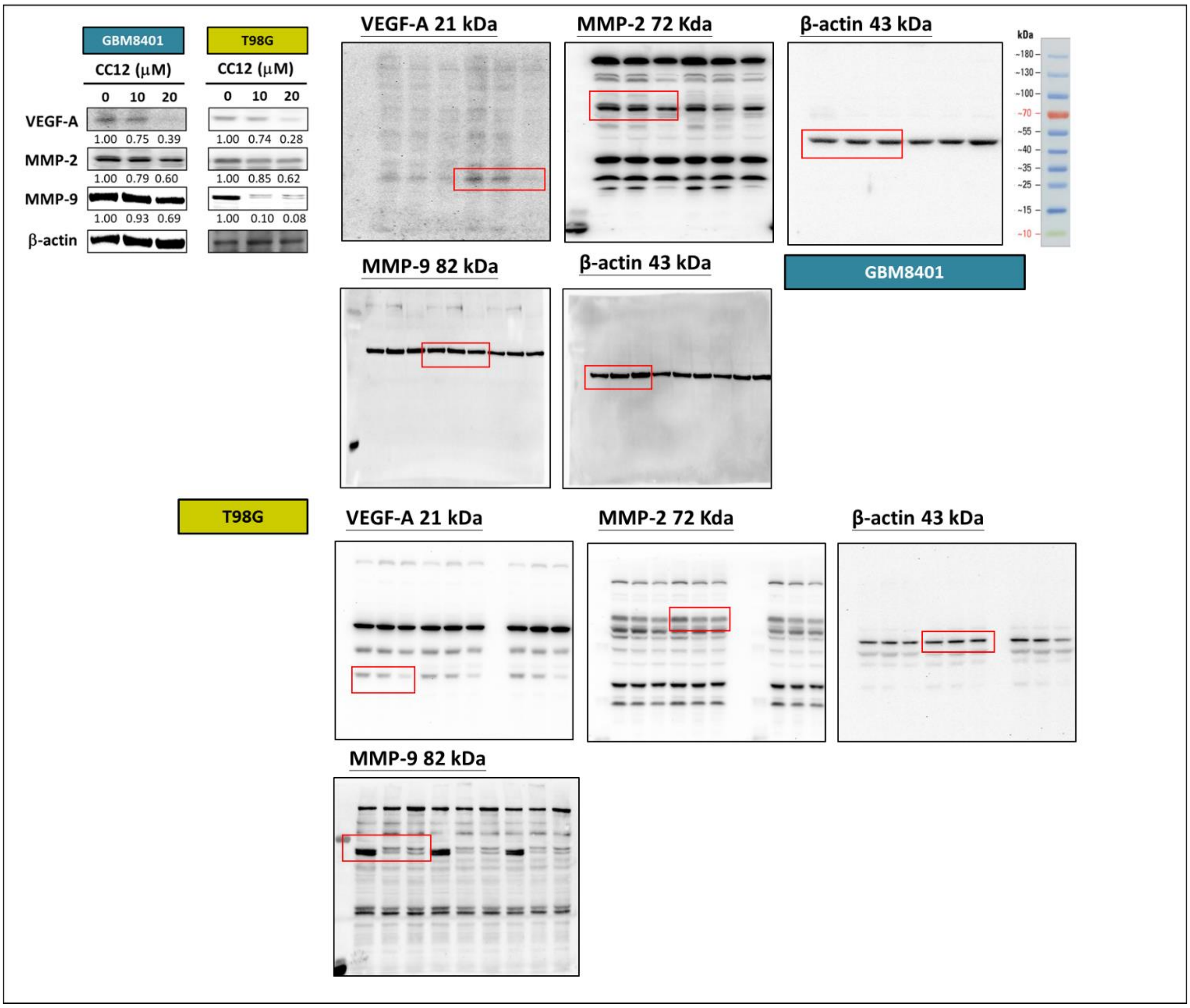

3.6 Full blot images of Figure 5E panel

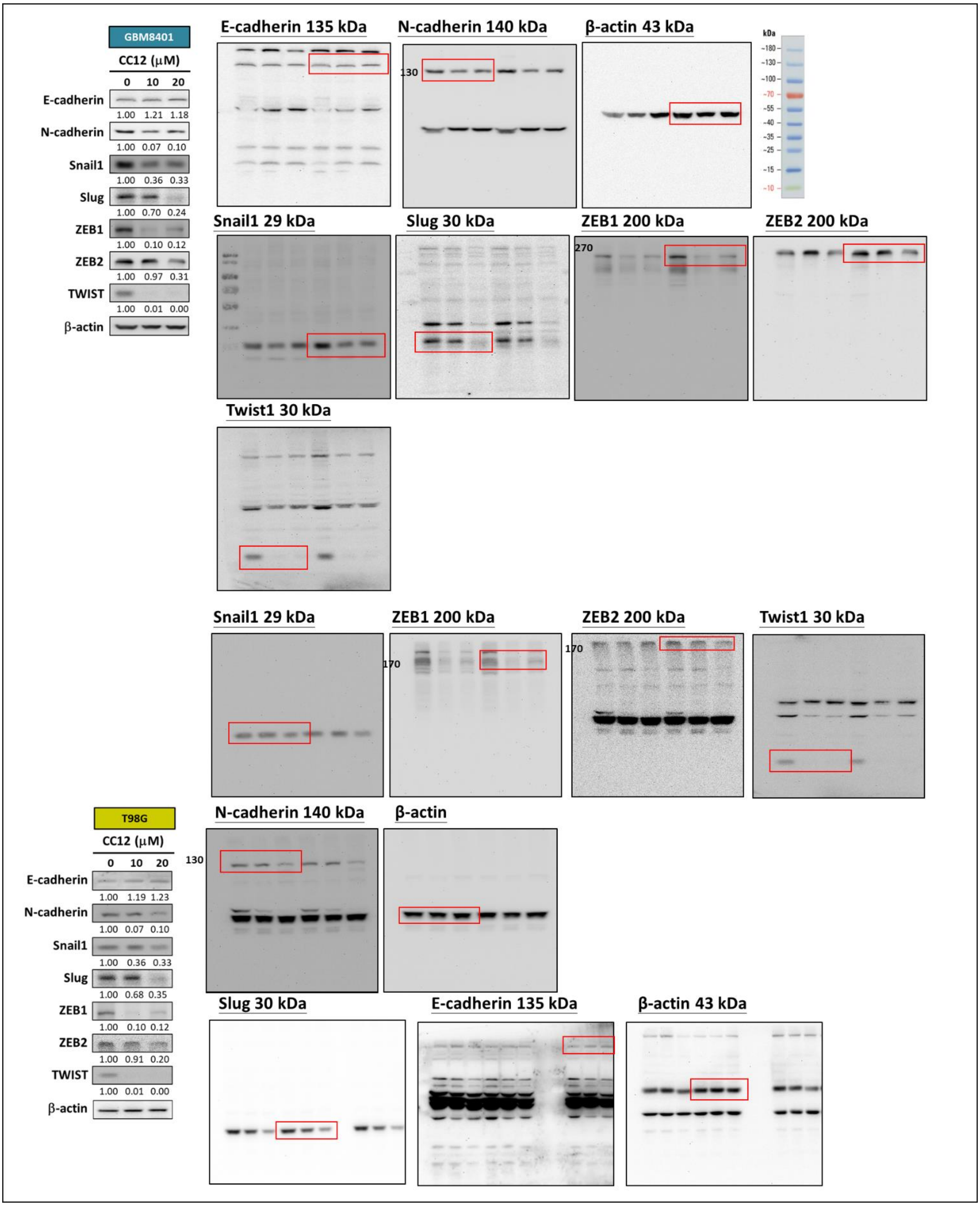

3.7 Full blot images of Figure 5F left panel

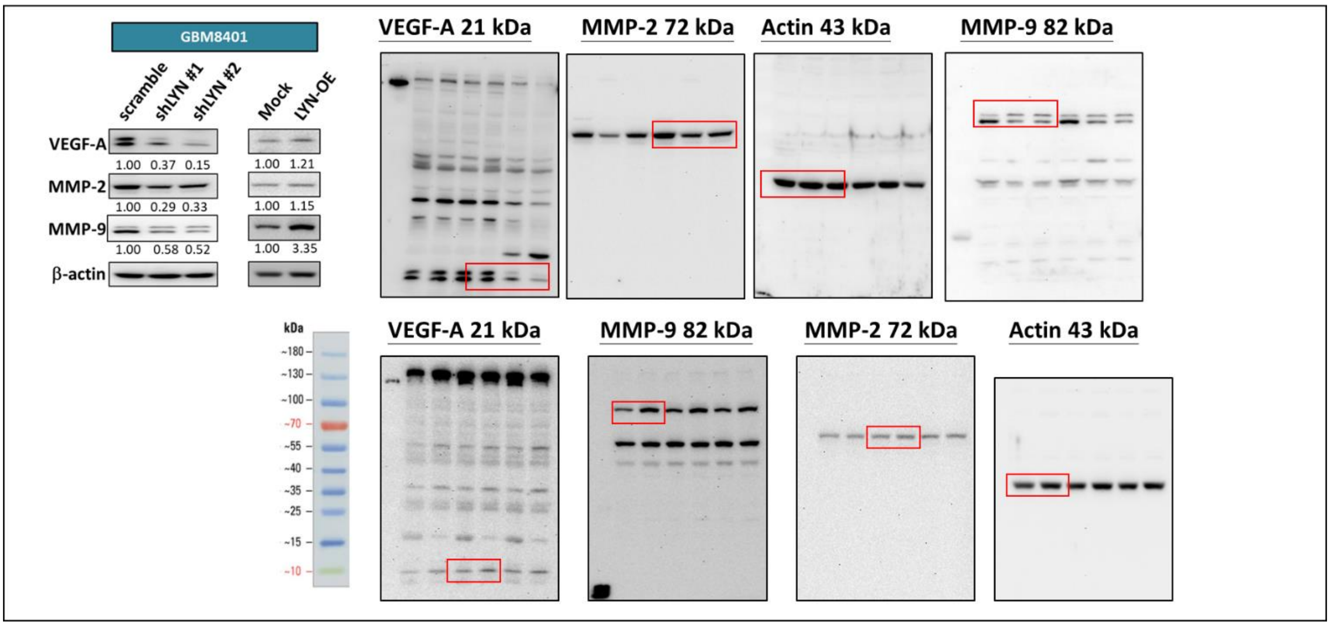

3.8 Full blot images of Figure 5F right panel

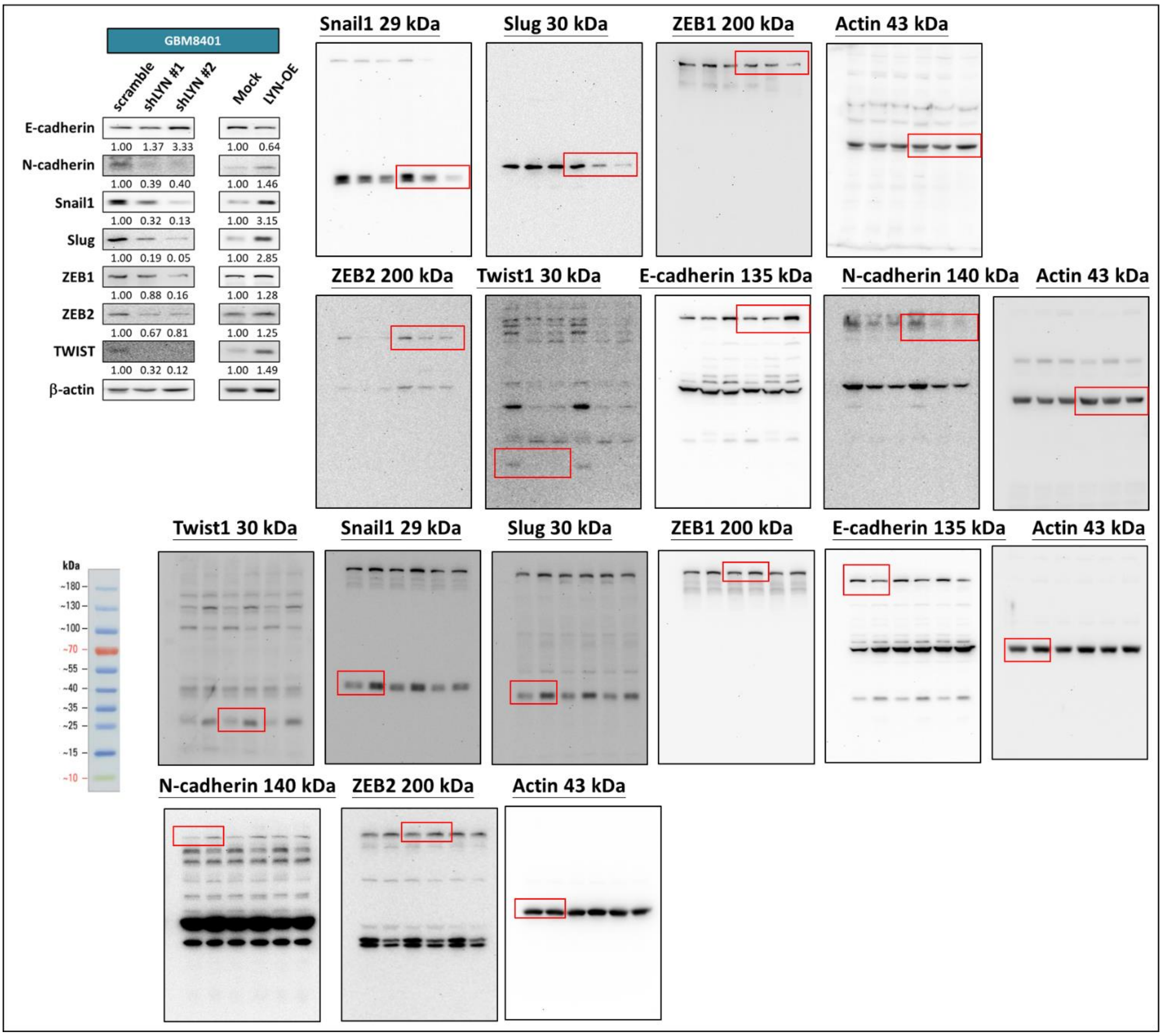

References

1. Chiang IT, Chen WT, Tseng CW, Chen YC, Kuo YC, Chen BJ, et al. Hyperforin Inhibits Cell Growth by Inducing Intrinsic and Extrinsic Apoptotic Pathways in Hepatocellular Carcinoma Cells. Anticancer Res. 2017; 37: 161-7.
2. Chiang CH, Chung JG, Hsu FT. Regorefenib induces extrinsic/intrinsic apoptosis and inhibits MAPK/NF-κB-modulated tumor progression in bladder cancer in vitro and in vivo. Environ Toxicol. 2019; 34: 679-88.
3. Yueh PF, Lee YH, Fu CY, Tung CB, Hsu FT, Lan KL. Magnolol Induces the Extrinsic/Intrinsic Apoptosis Pathways and Inhibits STAT3 Signaling-Mediated Invasion of Glioblastoma Cells. Life (Basel). 2021; 11.
4. Hsu FT, Chiang IT, Wang W-S. Induction of apoptosis through extrinsic/intrinsic pathways and suppression of ERK/NF-κB signalling participate in anti-glioblastoma of imipramine. J Cell Mol Med. 2020; 24: 3982-4000.
5. Hsu FT, Chiang IT, Kuo YC, Hsia TC, Lin CC, Liu YC, et al. Amentoflavone Effectively Blocked the Tumor Progression of Glioblastoma via Suppression of ERK/NF- κ B Signaling Pathway. Am J Chin Med. 2019; 47: 913-31.
6. Hsu FT, Liu HS, Ali AAA, Tsai PH, Kao YC, Lu CF, et al. Assessing the selective therapeutic efficacy of superparamagnetic erlotinib nanoparticles in lung cancer by using quantitative magnetic resonance imaging and a nuclear factor kappa-B reporter gene system. Nanomedicine. 2018; 14: 1019-31.
7. Huang YP, Ma YS, Kuo CL, Liao CL, Chen PY, Peng SF, et al. Demethoxycurcumin Suppresses Human Brain Glioblastoma Multiforme GBM 8401 Cell Xenograft Tumor in Nude Mice In Vivo. Int J Mol Sci. 2021; 22.
